# ﻿Unexpected species richness of the lichen genus *Protoblastenia* (Lecanorales, Psoraceae) in Finland

**DOI:** 10.3897/mycokeys.124.162802

**Published:** 2025-10-29

**Authors:** Juha Pykälä, Leena Myllys

**Affiliations:** 1 Nature Solutions, Finnish Environmental Institute, Latokartanonkaari 11, FI-00790 Helsinki, Finland Nature Solutions, Finnish Environmental Institute Helsinki Finland; 2 Botany and Mycology Unit, Finnish Museum of Natural History, P. O. Box 7, FI-00014 University of Helsinki, Helsinki, Finland University of Helsinki Helsinki Finland

**Keywords:** Calcareous rocks, DNA barcoding, Italy, ITS, lichenised fungi, new species, Norway, phylogeny

## Abstract

The taxonomy of *Protoblastenia* in Finland was studied, based on morphology and molecular data (nuITS rDNA sequences). Twenty species were recognised, with sixteen species being newly described here: *P. arupii***sp. nov.**, *P. borealis***sp. nov.**, *P. compressa***sp. nov.**, *P. dolomitica***sp. nov.**, *P. ekmanii***sp. nov.**, *P. fennoarctica***sp. nov.**, *P. minuta***sp. nov.**, *P. oulankaensis***sp. nov.**, *P. pseudocompressa***sp. nov.**, *P. pseudoterricola***sp. nov.**, *P. remota***sp. nov.**, *P. rikkinenii***sp. nov.**, *P. saanaensis***sp. nov.**, *P. timdalii***sp. nov.**, *P. violacea***sp. nov.** and *P. westbergii***sp. nov.** All species are confined to calcareous rocks, except *P. terricola* which also grows on calcareous soil. The calcareous fells in Enontekiö in NW Finland were identified as hot spots of *Protoblastenia* diversity. Nine newly-described species (*P. arupii*, *P. fennoarctica*, *P. minuta*, *P. pseudoterricola*, *P. rikkinenii*, *P. saanaensis*, *P. timdalii*, *P. violacea* and *P. westbergii*) are restricted to this area in Finland. Several of the species are semi-cryptic. On average, they may have minor morphological differences, but many specimens cannot be identified, based on morphology only. *Protoblastenia rikkinenii* is reported from Norway and *P. oulankaensis* from Italy, based on GenBank sequences. Full descriptions and a preliminary key of *Protoblastenia* in Finland are provided.

## ﻿Introduction

*Protoblastenia* (Zahlbr.) J. Steiner is a lichenised genus in the family Psoraceae. The genus includes ca. 15–25 accepted, mainly calcicolous, species (Castello and Nimis 1995; Kainz 2004; Kainz and Rambold 2004). Most species occur on calcareous rocks and a few on soil over calcareous rock.

Species of *Protoblastenia* have anthraquinones in the apothecia, causing predominantly orange-coloured discs. Other typical features are predominantly convex apothecia and pale, non-septate medium-sized spores (Kainz and Rambold 2004). *Protoblastenia* differs from the closely-related genus *Psora* Hoffm. in the absence or weakly developed upper cortex in the thallus and the absence of calcium oxalate crystals in the hypothecium (Timdal 1987; Kainz and Rambold 2004). Based on the phylogenetic studies of Kainz and Rambold (2004), Ekman and Blaalid (2011) and Miadlikowska et al. (2014), *Protoblastenia* forms a monophyletic group.

Kainz and Rambold (2004) studied the phylogeny and taxonomy of *Protoblastenia* in Central Europe. They ITS-sequenced 11 species: *Protoblastenia aurata* Poelt & Vězda, *P. calva* (Dicks.) Zahbr., *P. calvella* Kainz & Rambold, *P. cyclospora* (Hepp ex Körb.) Poelt, *P. incrustans* (DC.) J. Steiner, *P. laeta* (Poelt) Kainz & Rambold, *P. lilacina* Poelt & Vězda, *P. pseudoincrustans* ined., *P. rupestris* (Scop.) J. Steiner, *P. siebenhaariana* (Körb.) J. Steiner and *P. terricola* (Anzi) Lynge. Three European species, *Protoblastenia coniasis* (A. Massal.) Poelt (Poelt 1957), *P. geitleri* Zahlbr. (Zahlbruckner 1936) and *P. szaferi* J. Nowak (Nowak 1974), were not sequenced.

So far, seven species of *Protoblastenia* are known in Finland: *P. calva*, *P. calvella*, *P. incrustans*, *P. lilacina*, *P. rupestris*, *P. siebenhaariana* and *P. terricola* (Stenroos et al. 2016; Pykälä 2023). However, during the DNA barcoding of the Finnish lichen biota, it became apparent that the genus is much in need of revision. Here, we present the results of the revised taxonomy and show that the number of species in Finland is 20, almost three times more than previously reported.

## ﻿Material and methods

### ﻿Taxon sampling

This study is based on material collected by the first author (JP) during the years 1990–2022 in Finland. The specimens are stored in H.

### ﻿Morphology

Apothecia and thalli were hand-sectioned with razor blades. The sections were examined and measured in tap water. Asci and ascospores were also studied in squash preparations of apothecia mounted in water. Spores were measured using 10% potassium hydroxide (KOH). Additionally, the thickness of the hypothecium was examined by cutting the apothecia into two pieces and studying the pieces using a binocular microscope. The range of spore size is indicated as arithmetic mean and standard deviation. Minimum and maximum values are given in parentheses. The size of the apothecia (in diameter) is given in surface view.

### ﻿DNA extraction and sequencing

Total genomic DNA was extracted from the apothecia (1–3) of 1 – 31-year-old herbarium specimens.

We used two different techniques for extraction and sequencing. Most Finnish samples were sequenced as part of the “Finnish Barcode of Life” research project conducted from 2012–2023 (FinBOL, https://finbol.org/).

The samples were placed on 96-well microplates and sent to the Canadian Centre for DNA Barcoding (CCDB). CCDB’s standard protocols (documentation available at http://ccdb.ca/resources) were used for extraction, PCR and sequencing. The primers ITS1 and ITS4 (White et al. 1990) were used both for PCR and sequencing of the nuclear ribosomal ITS region. The barcode sequences and their trace files along with all relevant collection data and photographs of the voucher specimens were uploaded to the Barcode of Life Data Systems (BOLD, https://www.boldsystems.org) database.

DNA of the 31 specimens (Pykälä 5963, 8547, 29704, 33037, 34043, 35585, 39693, 39851, 39987, 41511b, 43105, 43194, 53252, 43340, 43524, 43605, 43624, 43855, 43910, 44115, 44130, 44177, 44275, 44314, 44431, 44438, 44876, 46413, 57501, 58392, 58457) was extracted using DNeasy® Blood & Tissue kits by Qiagen following the protocol described in Myllys et al. (2011). PCR reactions were prepared using PuReTaq Ready-To-Go PCR beads (GE Healthcare). The 25 µl reaction volume contained 19 µl of dH2O, 0.4 µM of each primer and 4 µl of extracted DNA. PCR was run under the following conditions: initial denaturation for 5 min at 95 °C followed by five cycles of 30 s at 95 °C (denaturation), 30 s at 58 °C (annealing) and 1 min at 72 °C (extension); in the remaining 35 cycles, the annealing temperature was decreased to 56 °C; the PCR schedule ended with a final extension for 7 min at 72 °C. PCR products were purified and sequenced by Macrogen Inc. (Amsterdam, the Netherlands; https://www.macrogen.com) or alternatively cleaned with ExoSAP (Affymetrix, Santa Clara, California, USA) and sequenced by FIMM Genomics (https://www2.helsinki.fi/en/infrastructures/genome-analysis/infrastructures/fimm-genomics). The primers ITS1F (Gardes and Bruns 1993) and ITS4 (White et al. 1990) were used both for PCR amplification and sequencing of the ITS regions.

### ﻿Phylogenetic analyses

All the ITS sequences of *Protoblastenia* available in GenBank (n = 55) were downloaded (1.3.2025). *Psora decipiens* (Hedw.) Hoffm. and *P. rubiformis* (Ach.) Hook. were used as outgroups, based on the phylogenies of Miadlikowska et al. (2014) and Evankow et al. (2025).

A total of 141 ITS sequences were aligned (Table 1) with MUSCLE v.3.8.31 (Edgar 2004) using EMBL-EBI’s freely available web service (http://www.ebi.ac.uk/Tools/msa/muscle/). The aligned dataset was subjected to Maximum Likelihood analysis (ML). The analysis was performed with RAxML v.8.1.15 (Stamatakis 2014) on the CSC – IT Center for Science server ((http://www.csc.fi). The ITS region was divided into three partitions: ITS1, 5.8S and ITS2. These partitions were analysed under the universal GTR-GAMMA model. Node support was estimated with 1000 bootstrap replications using the rapid bootstrap algorithm. Branches with bootstrap values ≥ 70% were considered strongly supported.

**Table 1.** Specimens used in the phylogenetic analyses. New sequences are in bold.

Taxon	Country	Voucher specimen	GenBank Accession number (ITS)
***Protoblastenia arupii***	**Finland**	**Pykälä 44130 (H)**	**PV766636**
*P. aurata*	Austria	Kainz 950 (M)	AY425658
*P. aurata*	Austria	Wittmann (M)	AY425659
***P. borealis***	**Finland**	**Pykälä 16567 (H)**	**PV766637**
***P. borealis***	**Finland**	**Pykälä 23710 (H)**	**PV766638**
***P. borealis***	**Finland**	**Pykälä 25292 (H)**	**PV766639**
***P. borealis***	**Finland**	**Pykälä 26097 (H)**	**PV766640**
***P. borealis***	**Finland**	**Pykälä 29704 (H)**	**PV766641**
***P. borealis***	**Finland**	**Pykälä 32757 (H)**	**PV766642**
***P. borealis***	**Finland**	**Pykälä 33037 (H)**	**PV766643**
***P. borealis***	**Finland**	**Pykälä 33101 (H)**	**PV766644**
***P. borealis***	**Finland**	**Pykälä 38784 (H)**	**PV766645**
***P. borealis***	**Finland**	**Pykälä 39151 (H)**	**PV766646**
***P. borealis***	**Finland**	**Pykälä 39942 (H)**	**PV766647**
***P. borealis***	**Finland**	**Pykälä 40695 (H)**	**PV766648**
***P. borealis***	**Finland**	**Pykälä 41355 (H)**	**PV766649**
***P. borealis***	**Finland**	**Pykälä 42802 (H)**	**PV766650**
***P. borealis***	**Finland**	**Pykälä 44641 (H)**	**PV766651**
***P. borealis***	**Finland**	**Pykälä 44906 (H)**	**PV766652**
***P. borealis***	**Finland**	**Pykälä 45759 (H)**	**PV766653**
***P. borealis***	**Finland**	**Pykälä 47217 (H)**	**PV766654**
***P. borealis***	**Finland**	**Pykälä 57501 (H)**	**PV766655**
***P. borealis***	**Finland**	**Pykälä 58196 (H)**	**PV766656**
***P. borealis***	**Finland**	**Pykälä 58392 (H)**	**PV766657**
***P. borealis***	**Finland**	**Pykälä 60511 & Kuusisto (H)**	**PV766658**
*P. calva*	Ireland	Sipman 30678 (B)	AY425628
*P. calva*	Austria	Kainz 981 (M)	AY425642
*P. calva*	Germany	Kainz 501 (M)	AY425643
*P. calva*	France	Rambold 6242 (M)	AY425644
*P. calva*	Germany	Kainz 852 (M)	AY425645
*P. calva*	Norway	Edvardsen & Ekman NO6 (BG)	EF524319
“*P. calva*”	Croatia	Gueidan CG659 (DUKE)	HQ650618
*P. calvella*	Germany	Kainz 764 (M)	AY425646
*P. calvella*	Germany	Kainz 765 (M)	AY425647
*P. calvella*	Austria	Kainz 967 (M)	AY425648
*P. calvella*	Germany	Kainz 805 (M)	AY425649
*P. calvella*	Finland	Pykälä 44807 (H)	OQ604661
*P. calvella*	Finland	Pykälä 43503 (H)	OQ604662
*P. calvella*	Sweden	Ekman & Eide Ekman 5821 (UPS)	OR773065
***P. compressa***	**Finland**	**Pykälä 35585 (H)**	**PV766659**
***P. compressa***	**Finland**	**Pykälä 36521 (H)**	**PV766660**
***P. compressa***	**Finland**	**Pykälä 38349 (H)**	**PV766661**
***P. compressa***	**Finland**	**Pykälä 38646 (H)**	**PV766662**
***P. compressa***	**Finland**	**Pykälä 38965 (H)**	**PV766663**
***P. compressa***	**Finland**	**Pykälä 41376 (H)**	**PV766664**
***P. compressa***	**Finland**	**Pykälä 41511b (H)**	**PV766665**
***P. compressa***	**Finland**	**Pykälä 42124 (H)**	**PV766666**
*P. cyclospora*	Italy	Tretiach (M)	AY425667
*P. cyclospora*	Greece	Kainz 913 (M)	AY425668
***P. dolomitica***	**Finland**	**Pykälä 36072 (H)**	**PV766667**
***P. dolomitica***	**Finland**	**Pykälä 39217 (H)**	**PV766668**
***P. dolomitica***	**Finland**	**Pykälä 39851 (H)**	**PV766669**
***P. dolomitica***	**Finland**	**Pykälä 43340 (H)**	**PV766670**
***P. dolomitica***	**Finland**	**Pykälä 43466 (H)**	**PV766671**
***P. dolomitica***	**Finland**	**Pykälä 43855 (H)**	**PV766672**
***P. dolomitica***	**Finland**	**Pykälä 44115 (H)**	**PV766673**
***P. ekmanii***	**Finland**	**Pykälä 37508 (H)**	**PV766674**
***P. fennoarctica***	**Finland**	**Pykälä 43105 (H)**	**PV766675**
***P. fennoarctica***	**Finland**	**Pykälä 43194 (H)**	**PV766676**
***P. fennoarctica***	**Finland**	**Pykälä 43656 (H)**	**PV766677**
***P. fennoarctica***	**Finland**	**Pykälä 44116 (H)**	**PV766678**
*P. incrustans*	Svalbard	Zhang	KP314370
*P. incrustans*	Svalbard	Zhang ZT2013200	KP314440
*P. incrustans*	Germany	Kainz 498 (M)	AY425630
*P. incrustans*	Germany	Kainz 855 (M)	AY425631
*P. incrustans*	Germany	Kainz 842 (M)	AY425632
*P. incrustans*	Germany	Kainz 228 (M)	AY425669
*P. incrustans*	Germany	Kainz 220 (M)	AY425670
*P. laeta*	Germany	Kainz 856 (M)	AY425650
*P. laeta*	Germany	Kainz 737 (M)	AY425651
*P. laeta*	Germany	Kainz 741 (M)	AY425652
*P. laeta*	France	Rambold 6253 (M)	AY425653
“*P. lilacina*”	Italy	Kainz 161 (M)	AY425629
*P. lilacina*	France	Sipman 22890 (B)	AY425660
*P. lilacina*	Germany	Kainz 809 (M)	AY425661
*P. lilacina*	Greece	Kainz 900 (M)	AY425662
*P. lilacina*	Germany	Kainz 788 (M)	AY425663
***P. lilacina***	**Finland**	**Pykälä 27009 (H)**	**PV766679**
***P. lilacina***	**Finland**	**Pykälä 27629 (H)**	**PV766680**
***P. lilacina***	**Finland**	**Pykälä 37012 (H)**	**PV766681**
***P. lilacina***	**Finland**	**Pykälä 57976 (H)**	**PV766682**
***P. minuta***	**Finland**	**Pykälä 44275 (H)**	**PV766683**
***P. oulankaensis***	**Finland**	**Pykälä 39629 (H)**	**PV766684**
***P. oulankaensis***	**Finland**	**Pykälä 39810 (H)**	**PV766685**
***P. oulankaensis***	**Finland**	**Pykälä 40201 (H)**	**PV766686**
***P. oulankaensis***	**Finland**	**Pykälä 44928 (H)**	**PV766687**
***P. oulankaensis***	**Finland**	**Pykälä 44949 (H)**	**PV766688**
***P. oulankaensis***	**Finland**	**Pykälä 44953 (H)**	**PV766689**
***P. oulankaensis***	**Finland**	**Pykälä 55995 (H)**	**PV766690**
***P. pseudocompressa***	**Finland**	**Pykälä 35901 (H)**	**PV766691**
***P. pseudocompressa***	**Finland**	**Pykälä 36325 (H)**	**PV766692**
***P. pseudocompressa***	**Finland**	**Pykälä 39367 (H)**	**PV766693**
***P. pseudocompressa***	**Finland**	**Pykälä 39693 (H)**	**PV766694**
***P. pseudocompressa***	**Finland**	**Pykälä 39987 (H)**	**PV766695**
***P. pseudocompressa***	**Finland**	**Pykälä 58457 (H)**	**PV766696**
*P. pseudoincrustans* ined.	Austria	Kainz 949 (M)	AY425664
*P. pseudoincrustans* ined.	Germany	Kainz 488 (M)	AY425665
*P. pseudoincrustans* ined.	Germany	Kainz 489 (M)	AY425666
***P. pseudoterricola***	**Finland**	**Pykälä 44157 (H)**	**PV766697**
***P. remota***	**Finland**	**Pykälä 8547 (H)**	**PV766698**
***P. remota***	**Finland**	**Pykälä 39455 (H)**	**PV766699**
***P. rikkinenii***	**Finland**	**Pykälä 43252 (H)**	**PV766700**
***P. rikkinenii***	**Finland**	**Pykälä 43910 (H)**	**PV766701**
***P. rikkinenii***	**Finland**	**Pykälä 44387 (H)**	**PV766702**
*P. rupestris*	Germany	Kainz 714 (M)	AY425654
*P. rupestris*	Germany	Kainz 791 (M)	AY425655
*P. rupestris*	Germany	Kainz 773 (M)	AY425656
*P. rupestris*	France	Rambold 6252 (M)	AY425657
*P. rupestris*	Norway	Edvardsen & Ekman NO8 (BG)	EF524318
*P. rupestris*	France	Gaya 415 & Guiedan (BCN)	EU639582
“*P. rupestris*”	Norway	Millanes (O)	KY266981
*P. rupestris*	Canada	McMullin (OAC)	KT695366
*P. rupestris*	Norway	Johnsen (BG)	MG926010
“*P. rupestris*”	UK	Cannon (K)	MZ159571
*P. rupestris*	Spain	Garrido-Benavent IGB1018 (VAL)	OQ550133
*P. rupestris*	USA	Lendemer 49650 (NY)	MK092337
***P. rupestris***	**Finland**	**Pykälä 5963 (H)**	**PV766703**
***P. rupestris***	**Finland**	**Pykälä 27569 (H)**	**PV766704**
***P. rupestris***	**Finland**	**Pykälä 34043 (H)**	**PV766705**
***P. rupestris***	**Finland**	**Pykälä 46413 (H)**	**PV766706**
“P. aff. rupestris”	USA	Leavitt SL19096	MZ922194
“P. aff. rupestris”	USA	Leavitt SL19099	MZ922195
***P. saanaensis***	**Finland**	**Pykälä 44431 (H)**	**PV766707**
***P. saanaensis***	**Finland**	**Pykälä 44438 (H)**	**PV766708**
*P. siebenhaariana*	Germany	Kainz 188 (M)	AY425627
*P. terricola*	Austria	Hafellner 42151 (GZU)	AY425639
*P. terricola*	Austria	Kainz 977 (M)	AY425640
*P. terricola*	Switzerland	Sippman 30291 (B)	AY425641
***P. terricola***	**Finland**	**Pykälä 44876 (H)**	**PV766709**
***P. timdalii***	**Finland**	**Pykälä 43624 (H)**	**PV766710**
***P. timdalii***	**Finland**	**Pykälä 44177 (H)**	**PV766711**
***P. violacea***	**Finland**	**Pykälä 43015 (H)**	**PV766712**
***P. violacea***	**Finland**	**Pykälä 43524 (H)**	**PV766713**
***P. violacea***	**Finland**	**Pykälä 43605 (H)**	**PV766714**
***P. westbergii***	**Finland**	**Pykälä 43196 (H)**	**PV766715**
***P. westbergii***	**Finland**	**Pykälä 43279 (H)**	**PV766716**
***P. westbergii***	**Finland**	**Pykälä 44314 (H)**	**PV766717**
***P. westbergii***	**Finland**	**Pykälä 44316 (H)**	**PV766718**
*Psora rubiformis*	Norway	Timdal (O)	KY266957
*P. decipiens*	Greenland	Timdal 10078 (O)	EF524326

## ﻿Results and discussion

**Figure 1.**
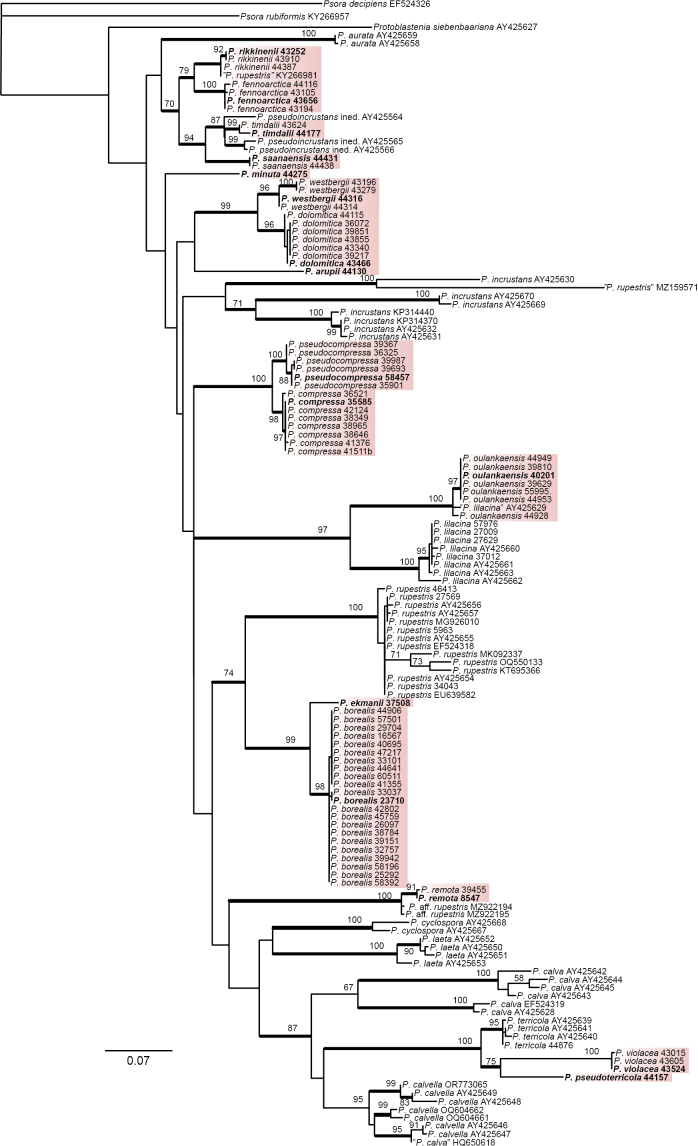
Phylogenetic relationships of the studied *Protoblastenia* species, based on a Maximum Likelihood (ML) analysis of the ITS dataset. Maximum likelihood bootstrap values > 50% are shown at nodes. Thickened lines indicate bootstrap values > 70%. New species described in this study are indicated with shadowed boxes. GenBank accession numbers for sequences downloaded from GenBank and collection numbers for the specimens sequenced for this study are shown after the taxon names. Holotype specimens of the new species are shown in bold.

We generated 83 new ITS sequences obtained from the Finnish *Protoblastenia* specimens. In the ITS phylogeny (Fig. 1), the sequenced Finnish specimens were divided into 20 lineages of which 16 do not match any described species. These lineages, when represented by multiple samples, received high support values (96–100%) and are described here as new species. Typically, infraspecific differences in ITS in the Finnish *Protoblastenia* species are small.

As expected, the phylogeny, based on only ITS sequences, remains unsupported at deeper nodes. However, six well-supported (ML bootstrap support > 70%) lineages represented by more than one species were identified: 1) *P. fennoarctica* sp. nov. + *P. rikkinenii* sp. nov. + *P. saanaensis* sp. nov. + *P. timdalii* sp. nov. + *P. pseudoincrustans* ined. 2) *P. dolomitica* sp. nov. + *P. westbergii* sp. nov., 3) *P. compressa* sp. nov. + *P. pseudocompressa* sp. nov., 4) *P. lilacina* + *P. oulankaensis* sp. nov., 5) *P. borealis* sp. nov. + *P. ekmanii* sp. nov. + *P. rupestris*, 6) *P. calva* + P. *calvella* + *P. pseudoterricola* sp. nov. + *P. terricola* + *P. violacea* sp. nov.

All the new Finnish species with more than one sequence show a barcode gap, i.e. their genetic distance to the nearest neigbour exceeds their maximum intraspecific distance (Table 2). Note that the gap is slightly smaller if GenBank sequences here identified as *P. oulankaensis* and *P. remota* are included. The maximum genetic distance within the species including the Italian sequence of *P. oulankaensis* (AY425629) is 1.7%. If the two North American sequences (MZ922194, MZ922195) are included in *P. remota*, the maximum genetic distance of *P. remota* is 1.6%.

**Table 2.** Genetic distances within and between the new species of *Protoblastenia*, based on the Finnish and GenBank sequences.

	Maximum intraspecific distance (%)	Minimum distance to nearest neighbour (%)	Barcodegap (%)	Nearest neighbour
*P. arupii* (n = 1)		9.9		*P. dolomitica*
*P. borealis* (n = 22)	0.6	2.5	1.9	*P. ekmanii*
*P. compressa* (n = 8)	0.8	2.0	1.2	*P. pseudocompressa*
*P. dolomitica* (n = 7)	1.1	3.9	2.8	*P. westbergii*
*P. ekmanii* (n = 1)		2.5		*P. borealis*
*P. fennoarctica* (n = 4)	0.8	4.2	3.4	*P. rikkinenii*
*P. minuta* (n = 1)		9.2		*P. timdalii*
*P. oulankaensis* (n = 7)	1.2	9.6	8.4	*P. lilacina*
*P. pseudocompressa* (n = 6)	0.6	2.0	1.4	*P. compressa*
*P. pseudoterricola* (n = 1)		4.9		*P. terricola*
*P. remota* (n = 2)	0.4	14.0	13.6	*P. incrustans*
*P. rikkinenii* (n = 3)	0.6	4.4	3.8	*P. fennoarctica*
*P. saanaensis* (n = 2)	0.2	5.2	5.0	*P. timdalii*
*P. timdalii* (n = 2)	0.7	2.8	2.1	*P. pseudoincrustans* ined.
*P. violacea* (n = 3)	0.2	6.9	6.7	*P. terricola*
*P. westbergii* (n = 4)	2.2	4.6	2.4	*P. dolomitica*

The specimens previously treated as *P. siebenhaariana* by Stenroos et al. (2016) belong to six different species, all characterised by a violet hypothecium: *Protoblastenia arupii* sp. nov., *P. dolomitica* sp. nov., *P. pseudoterricola* sp. nov., *P. terricola*, *P. violacea* sp. nov. and *P. westbergii* sp. nov. However, all these species have more densely occurring apothecia than *P. siebenhaariana*. Furthermore, the specimens previously identified as *P. incrustans* belong to *P. compressa* sp. nov. and *P. pseudocompressa* sp. nov.

Specimens treated as *Protoblastenia rupestris* belong in nine species: *P. rupestris*, *P. calvella*, *P. borealis* sp. nov., *P. fennoarctica* sp. nov., *P. minuta* sp. nov., *P. remota* sp. nov., *P. rikkinenii* sp. nov., *P. saanaensis* sp. nov. and *P. timdalii* sp. nov. The sequenced specimens previously identified as *P. calva* belong to *P. lilacina*, *P. oulankaensis* sp. nov. and *P. pseudocompressa*. Previously only Finnish specimens on soil have been identified as *P. terricola*, but the species is also epilithic in Finland.

Unfortunately, sequences of *P. coniasis*, *P. geitleri* and *P. szaferi* were not available in GenBank. However, all three species differ morphologically from our new species: *Protoblastenia coniasis* resembles *P. incrustans*, but has a yellow thallus that gradually turns brownish-rose in the herbarium (Poelt 1957). *Protoblastenia geitleri* has globose spores (Zahlbruckner 1936), a feature not present in any of the Finnish species. *Protoblastenia szaferi* is characterised by an endolithic thallus, mainly flat apothecia immersed in rock and relatively distinct apothecial margins (Hafellner 2006). Such a combination of morphological characteristics was not observed in the Finnish material.

The number of new species is surprisingly high, particularly compared to the global number of accepted species. Even though the new species are genetically distinct, their morphological identification may be difficult. On average, all species may differ morphologically, but for several species, more material is needed to confirm this. In addition, there is considerable overlap in morphological characteristics between the species. Most of the species may be semi-cryptic, a feature that may be common in lichens (e.g. Orange (2012); Pykälä et al. (2020)). Some specimens can be identified by morphology, while identification of other specimens remains uncertain. Table 3 presents the most important apothecial characters between the new species.

**Table 3.** The most important apothecial characters between the new species of *Protoblastenia*.

	Mean spore length and width (μm)	Size of hypothecium (min and max) (μm)	Width of apothecia (min and max) (mm)
*P. arupii*	12.8 × 6.8	50–60	0.2–0.5
*P. borealis*	11.2 × 6.2	40–160	0.2–1.0
*P. compressa*	11.4 × 5.9	40–100	0.2–0.8
*P. dolomitica*	12.8 × 5.4	40–100	0.15–0.7
*P. ekmanii*	10.2 × 5.9	120–200	0.4–0.8
*P. fennoarctica*	10.0 × 5.3	60–280	0.3–0.9
*P. minuta*	7.9 × 4.5	70–100	0.2–0.4
*P. oulankaensis*	13.9 × 7.4	60–220	0.3–0.9
*P. pseudocompressa*	10.6 × 5.5	50–120	0.2–0.7
*P. pseudoterricola*	9.9 × 5.5	60–100	0.3–0.8
*P. remota*	11.4 × 5.6	100–200	0.2–0.6
*P. rikkinenii*	9.6 × 5.1	80–300	0.3–0.8
*P. saanaensis*	8.4 × 4.8	40–100	0.3–0.5
*P. timdalii*	9.2 × 4.8	60–100	0.3–0.8
*P. violacea*	9.2 × 4.6	100–280	0.3–1.0
*P. westbergii*	12.0 × 6.1	60–150	0.2–1.1

Numerous studies have shown that DNA barcoding is efficient in species delimitation and in finding morphologically cryptic or semi-cryptic species (e.g. Divakar et al. (2016); Lücking et al. (2020a); Zhang et al. (2022); Printzen et al. (2023)). However, in several lichen groups, lack of resolution in recently evolving species complexes may occur (Lücking et al. 2020a, b). Thus, lineages receiving high support values in the ITS phylogeny and showing a barcode gap can be described as new species even if they are morphologically cryptic or semi-cryptic. Our results show that ITS is well suitable for species delimitations of *Protoblastenia*.

The calcareous fells in Enontekiö in NW Finland were identified as hot spots of *Protoblastenia* diversity. Nine newly-described species are restricted to this area in Finland. The importance of the area for rare lichens has been previously shown in several studies (see, for example, Pykälä (2010); Pykälä et al. (2017); Pykälä and Lommi (2021)).

### ﻿Taxonomy

The species descriptions are based on the sequenced specimens collected in Finland. All specimens are deposited in H. The ITS sequences of only four species present in GenBank match the Finnish specimens: *Protoblastenia calvella*, *P. lilacina*, *P. rupestris* and *P. terricola*. However, *P. calvella* may be heterogeneous. More material is needed before any taxonomic conclusions can be made. No specimens whose barcode matched GenBank specimens of *P. calva*, *P. incrustans* or *P. siebenhaariana* were found. The Finnish specimens also differ from the morphological descriptions of these species (Kainz and Rambold 2004; Cannon et al. 2022). Thus, these species have been deleted from the Finnish lichen biota. Based on ITS sequences, *P. calva* in GenBank may be divided into two species: one sequenced from Central Europe, the other from Ireland and Norway. Similarly, in our phylogeny, the cited GenBank sequences of *P. incrustans* seem to represent three species.

#### ﻿ Protoblastenia arupii

Taxon classificationFungiLecanoralesPsoraceae

Pykälä & Myllys sp. nov.

FABBEBBC-9C46-543F-B64C-AC4C9EA452F3

859623

Fig. 2A

##### Diagnosis.

Differs from *P. dolomitica* and *P. westbergii* in having only a slightly rimose thallus.

##### Type.

Finland • Enontekiön Lappi, Enontekiö, Kilpisjärvi, Saana, fell, steep NE-slope, dolomite rock outcrop, on NE-facing wall, 820 m a.s.l., 69°02'N, 20°51'E, 11 August 2011, J. Pykälä 44130 (H9250904 – holotype, GenBank accession number: PV766636).

##### Description.

Thallus grey, slightly rimose, ca. 0.02–0.05 mm thick, K-, UV-. Apothecia orange, 0.2–0.5 mm, slightly convex to convex, 1/2-immersed in thallus; ca. 60 apothecia / cm^2^. Epihymenium brown-yellow to orange-brown, 12–15 μm thick, K+ violet. Hymenium 50–60 μm thick. Hypothecium red-violet, ca. 50–60 μm thick. Paraphyses ca. 1.5–2 μm thick, apex not thickened. Ascospores 0-septate, (10.3–)11.8–12.8–13.8(–15.1) × (5.3–)5.7–6.8–7.8(–9.4) μm (n = 27).

**Figure 2.**
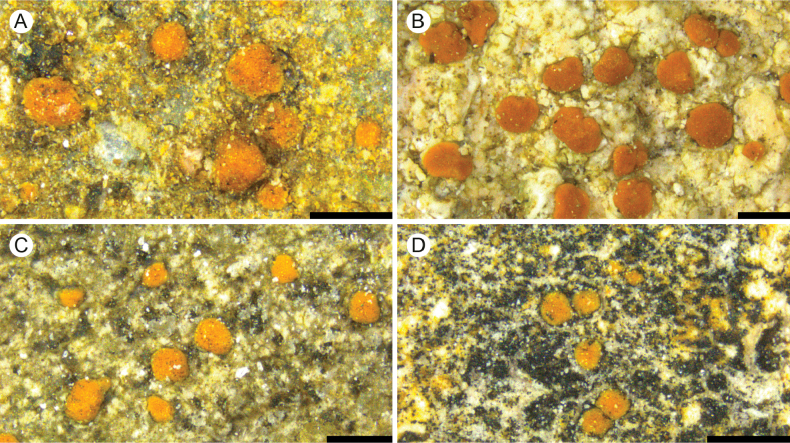
A. *Protoblastenia arupii* (holotype); B. *P. borealis* (holotype); C. *P. compressa* (holotype); D. *P. dolomitica* (holotype). Scale bars: 0.5 mm.

##### Habitat and distribution.

Only one specimen has been found on the calcareous Saana fell in NW Finland. The species was associated with *Polyblastia* sp. and *Rhizocarpon petraeum* (Wulfen) A. Massal.

##### Etymology.

The specific epithet is in honour of Dr Ulf Arup (Lund) for his many important contributions on the taxonomy (particularly Teloschistaceae and Lecanoraceae) and biogeography of especially Fennoscandian lichens.

##### Notes.

*Protoblastenia arupii* is related to *P. dolomitica* and *P. westbergii* in the ITS tree, although the relationship remains unsupported. The only known specimen is characterised by a thin red-violet hypothecium and large spores, some of them particularly broad. *Protoblastenia dolomitica* and *P. westbergii* differ in their rimose to areolate thallus and slightly narrower spores. An ITS marker as a DNA barcode may be needed for unambiguous identification.

#### ﻿ Protoblastenia borealis

Taxon classificationFungiLecanoralesPsoraceae

Pykälä & Myllys sp. nov.

9C8CA1D6-2BD6-50B2-A2EE-4956005B2AA6

859624

Fig. 2B

##### Diagnosis.

Differs from *P. rupestris* in having a mostly rimose thallus.

##### Type.

Finland • Varsinais-Suomi, Lohja, Lylyinen, Sääksniemi, N of summer cottages, small calcareous rock outcrop, on SW-slope, 50 m a.s.l., 60°15'N, 23°57'E, 16 September 2003, J. Pykälä 23710 (H9250905 – holotype, UPS – isotype, GenBank accession number: PV766638).

##### Description.

Thallus white to pale grey, endolithic (rarely), rimose (usually) to areolate, areoles 0.1–0.8 mm, ca. 0.02–0.2 mm thick, K-, C-, UV- to UV+ bluish-white, algal cells 5–12(–17) μm. Apothecia yellow-orange to dirty brown-orange, 0.2–1.0 mm, slightly convex to strongly convex, ½-immersed in thallus to superficial, occasionally leaving shallow pits, K+violet; ca. 30–140 apothecia / cm^2^. Epihymenium orange-brown, 10–18 μm thick, K+ violet. Hymenium 50–90(–100) μm thick. Hypothecium colourless to pale yellow, rarely lower part reddish-brown, occasionally with oil droplets, ca. 40–160 μm thick. Paraphyses ca. (1.5–)2–2.5 μm thick, apex not or slightly thickened, sparingly branched and anastomosing. Ascospores 0-septate, (7.6–)9.6–11.2–12.7(–16.1) × (4.8–)5.4–6.2–7.0(–7.7) μm (n = 146).

##### Habitat and distribution.

This species is the most common species of *Protoblastenia* in Finland. It occurs from the hemi-boreal zone to middle Lapland. It grows on calcareous and calciferous rocks on both sun-exposed and shady habitats, but may prefer the latter. It also often grows in lime quarries. Specimens from concrete probably also belong in this species, but no sequences of them are available. Typical companion species include, for example, *Acarospora glaucocarpa* (Ach.) Körb., *Clauzadea monticola* (Schaer.) Hafellner & Bellem., *Farnoldia jurana* (Schaer.) Hertel, *Lathagrium fuscovirens* (With.) Otálora, P. M. Jørg. & Wedin, *Phaeophyscia sciastra* (Ach.) Moberg, *Scytinium parvum* (Degel.) Otálora, P. M. Jørg. & Wedin, *Verrucaria deversa* Vain., *V. epilithea* Vain., *V. muralis* Ach. and *V. nigrescens* Pers.

##### Etymology.

The name refers to the geographic distribution of the species in the boreal vegetation zone.

##### Notes.

*Protoblastenia borealis* is morphologically very variable and may differ in the size and density of apothecia and in the thickness of the hypothecium. The sister species *Protoblastenia ekmanii* is morphologically similar, but appears to differ in having a thicker hypothecium. The new species is also difficult to distinguish from the closely-related *P. rupestris*. However, *P. rupestris* does not have any strongly convex apothecia and has, on average, a thicker thallus, which is mostly areolate. *Protoblastenia pseudocompressa* often/usually has plane apothecia, but some specimens with convex apothecia are somewhat similar to *P. borealis*. The two Finnish specimens of *Protoblastenia calvella* resemble *P. borealis* in morphology; they have strongly convex apothecia and a thick hypothecium which were also observed in a few *P. borealis* specimens. Due to these overlaps, ITS is needed for unambiguous identification of *P. borealis*.

##### Other sequenced specimens.

Finland • Varsinais-Suomi, Karkkila, Haavisto, Herneojankallio, pyroxene gneiss rock outcrop, on high SW-facing overhanging wall, scarce, 80 m a.s.l., 60°29'N, 24°20'E, 18.5.1996, J. Pykälä 16567 (H); • Lohja, Torhola, 300 m N-NE of Kallioranta, young pine plantation, S-slope, almost 1 m high rock wall of calcareous rock outcrop, rather scarce, 60 m a.s.l., 60°15'N, 23°52'E, 22.7.2004, J. Pykälä 25292 (H); • Karjalohja, Karkali, 200 m SW Karkali farm, on a shore of Lohjanjärvi Lake, small calcareous rock outcrop, on stone, 33 m a.s.l., 60°14'N, 23°49'E, 19.10.2004, J. Pykälä 26097 (H); • Pohja, Kuovila, 300 m SW of Vihreämäki, abandoned lime quarry, NE-facing wall, 37 m a.s.l., 60°08'N, 23°24'E, 12.10.2006, J. Pykälä 29704 (H); • Suomusjärvi, Sallittu, Huuttavanmäki, calciferous rock outcrop, on W-facing wall, 110 m a.s.l., 60°18'N, 23°37'E, 28.6.2008, J. Pykälä 32757 (H); • Särkisalo, Förby, E of Vähämaankaula, abandoned lime quarry, on N-facing wall, 7 m a.s.l., 23.7.2008, J. Pykälä 33037 (H); • Särkisalo, Förby, E of Vähämaankaula, abandoned lime quarry, on NW-facing wall, 7 m a.s.l, 23.7.2008, J. Pykälä 33101 (H); • Kemiönsaari (Västanfjärd), Finsjö, Verkviksudden, calcareous rock outcrop, on 1.2 m high E-facing wall, 5 m a.s.l., 60°00'N, 22°41'E, 13.7.2010, J. Pykälä 38784 (H); • Salo (Suomusjärvi), Hinttala, W of Kalattomansuo, *Picea abies*-dominated herb-rich forest, abandoned lime quarry, on S-facing wall, 110 m a.s.l, 60°23'N, 23°39'E, 28.9.2010, J. Pykälä 41355 (H); • Etelä-Savo, Kerimäki, Ruokojärvi, Pitkäniemi, close by abandoned lime quarry, young *Pinus sylvestris*-forest, on calcareous boulder, 90 m a.s.l., 61°56'N, 29°00'E, 15.9.2011, J. Pykälä 45759 (H); • Pohjois-Karjala, Juankoski, Siikajärvi, Huosiaisniemi, Nature Reserve, mixed herb-rich forest, dolomite rock outcrop, on W-facing wall, scarce, 100 m a.s.l., 63°12'N, 28°21'E, 25.7.2011, J. Pykälä 42802 (H); • Juankoski, Siikajärvi, Lauantaijoki, Kalliola 250 m N, calciferous serpentine rock outcrop, on NW-facing wall, 132 m a.s.l., 63°09'N, 28°36'E, 9.9.2013, J. Pykälä 47217 (H); • Keski-Pohjanmaa, Vimpeli, Sääksjärvi, Huosianmaankallio, lime quarry, quarry spoil, on calcareous boulder, rather scarce, 135 m a.s.l., 63°09'N, 24°04'E, 1.9.2010, J. Pykälä 40695 (H); • Kainuu, Hyrynsalmi, Moisiovaara, Hyyryläinen SW, *Pinus sylvestris*-dominated heath forest, VMT site type, calciferous serpentine rock outcrop, on 1 m high W-facing wall, 190 m a.s.l., 64°32'N, 29°05'E, 30.5.2021, J. Pykälä 57501 (H); • Koillismaa, Kuusamo, Oulanka National Park, Kiutaköngäs 400 m N, *Pinus sylvestris* - herb-rich forest, small dolomite rock outcrop, on SE-slope, rather scarce, 175 m a.s.l., 66°22'N, 29°19'E, 3.8.2010, J. Pykälä 39151; • Kuusamo, Juuma, Oulanka National Park, Hautaniitynvuoma, gorge, calciferous (dolomite) schistose rock outcrop, on SW-facing wall, 195 m a.s.l, 66°15'N, 29°27'E, 14.8.2010, J. Pykälä 39942 (H); • Kuusamo, Juuma, Oulanka National Park, Hautaniitynvuoma, gorge, NE-slope, calciferous (dolomite) schistose rock outcrop, on NE-facing wall, 179 m a.s.l., 66°15'N, 29°26'E, 21.8.2011, J. Pykälä 44641 (H); • Salla, Oulanka National Park, Savilampi 1.4 km NE, bank of Savinajoki River, cliff, dolomite rock outcrop, on SE-facing wall, 185 m a.s.l., 66°26'N, 29°11'E, 23.8.2011, J. Pykälä 44906 (H); • Kuusamo, Kallunki, Juhonlampi, young *Pinus sylvestris*-dominated heath forest, calcareous rock outcrop, SW-slope, on 80 cm high SW-facing wall, 236 m a.s.l., 66°22'N, 29°03'E, 5.8.2021, J. Pykälä 58196 (H); • Kuusamo, Vasaraperä, Havukkalampi N, mixed herb-rich heath forest, calcareous rock outcrop, on 100 cm high SW-facing wall, 246 m a.s.l., 66°06'N, 28°44'E, 8.8.2021, J. Pykälä 58392 (H); • Kittilän Lappi, Kittilä, Tepsa, Herravaara SE, clear-cut heath forest, small calcareous rock outcrop, on 1.5 m high SE-facing wall, scattered, 15.9.2022 J. Pykälä & I. Kuusisto 60511 (H).

#### ﻿ Protoblastenia calvella

Taxon classificationFungiLecanoralesPsoraceae

Kainz & Rambold, Bibl. Lichenol. 88: 290 (2004)

F2FB6207-636D-5608-A655-A6F95C6B5CAC

##### Description.

Thallus white to whitish-grey, rimose to areolate, areoles 0.2–0.5 mm, ca. 0.02–0.2 mm thick, K-, C-, UV-, algal cells 5–8 μm. Apothecia orange-yellow to orange, 0.3–0.6 mm, strongly convex, superficial, not leaving pits to leaving shallow pits, K+violet; ca. 40–100 apothecia /cm^2^. Epihymenium dark orange-brown, 12–15 μm thick, K+ violet. Hymenium 80–100 μm thick. Hypothecium colourless to pale yellow, ca. 100–180 μm thick. Paraphyses ca. 2–2.5 μm thick, apex slightly thickened, often branching. Ascospores 0-septate, (7.6–)9.4–10.7–12.0(–12.7) × (4.4–)5.2–5.9–6.7(–7.8) μm (n = 29).

##### Habitat and distribution.

Only two specimens are known from northern Finland (Pykälä 2023). The habitats are sun-exposed calcareous and calciferous rock outcrops where the species grows on rock or on pebbles. The species is associated with *Enchylium polycarpon* (Hoffm.) Otálora, P. M. Jørg. & Wedin and *Rhizocarpon petraeum*.

##### Notes.

In the ITS phylogeny, the *P. calvella* specimens are divided into three strongly-supported lineages: the first one includes three specimens collected in Austria, Germany and Sweden; the second group includes two Finnish specimens and the third group consists of specimens collected in Croatia and Germany (Fig. 1). More material is needed to determine whether *P. calvella* represents several species. *Protoblastenia calvella* is a cryptic species which cannot be identified without molecular data (Wirth et al. 2013). Older apothecia are often brown-orange to dark red-brown (Svensson et al. 2024), but such colouration was not observed in the Finnish specimens. It may be confused with several other *Protoblastenia* species. A few specimens of *P. borealis* have a thick hypothecium and/or hymenium and thus resemble the Finnish specimens of *P. calvella*. The Finnish specimens of *P. rupestris* have a thinner hymenium and hypothecium compared to *P. calvella*. Furthermore, the apothecia tend to be predominantly orange, while in *P. calvella*, they are orange yellow. *Protoblastenia ekmanii* may differ in having darker orange apothecia. *Protoblastenia fennoarctica* can be rather similar to *P. calvella*, but its apothecia vary from plane to strongly convex and its hymenium is thinner (ca. 50–80 μm thick).

##### Specimens examined.

• Enontekiön Lappi, Enontekiö, Porojärvet, Toskalharji, Toskalpahta, fell, dolomite rock outcrop, on steep SW-slope, 820 m a.s.l., 69°11'N, 21°30'E, 4.8.2011, J. Pykälä 43503 (H); • Koillismaa, Kuusamo, Juuma, Niskakoski, river bank, calciferous (dolomite) schistose rock outcrop, on pebbles, 224 m a.s.l., 66°16'N, 29°24'E, 22.8.2011, J. Pykälä 44807 (H).

#### ﻿ Protoblastenia compressa

Taxon classificationFungiLecanoralesPsoraceae

Pykälä & Myllys sp. nov.

05B49C28-0BF1-579B-B752-80480A65D4C3

859625

Fig. 2C

##### Diagnosis.

Differs from *P. pseudocompressa* in having plane to slightly convex apothecia.

##### Type.

Finland • Varsinais-Suomi, Parainen (Korppoo), Elfsjö, Hummelskär Island, close by shore of the Baltic Sea, calcareous rock outcrop, on shady 1 m high NE-facing wall, 5 m a.s.l., 60°08'N, 21°24'E, 31 July 2009, J. Pykälä 35585 (H9250940 – holotype, GenBank accession number: PV766659).

##### Description.

Thallus white to pale grey, continuous, rimose to rarely areolate, areoles 0.2–0.8 mm, ca. 0.01–0.15 mm thick, K-, C-, UV- to UV+ bluish-white, algal cells 4–9 μm. Apothecia yellow, yellow-orange to orange, 0.2–0.8 mm, plane to slightly convex, 1/4–1-immersed in thallus, occasionally leaving shallow pits, K+ violet; ca. 20–190 apothecia / cm^2^. Epihymenium orange-brown, 12–18 μm thick, K+ violet. Hymenium 50–60 μm thick. Hypothecium colourless to yellow, ca. 40–100 μm thick. Paraphyses ca. 2–2.5 μm thick, apex not thickened to slightly thickened, sparingly branched and anastomosing. Ascospores 0-septate, (8.6–)9.8–11.4–12.6(–13.5) × (4.8–)5.1–5.9–6.7(–7.4) μm (n = 45).

##### Habitat and distribution.

This species has ca. 40 known localities in the hemi-boreal vegetation zone in SW Finland (seven localities confirmed by ITS). It grows on calcareous rocks and in lime quarries, often on N-facing walls. It seems to prefer shady habitats. In the SW archipelago of Finland, *P. compressa* may be the most common *Protoblastenia* species. Typical companion species are *Bagliettoa baldensis* (A. Massal.) Vězda s.lat, *Clauzadea monticola*, *Lepraria finkii* (B. de Lesd.) R. C. Harris, *Polyblastia abscondita* (Nyl.) Arnold, *Sagiolechia protuberans* (Ach.) A. Massal., *Thelidium decipiens* (Nyl.) Kremp., *T. incavatum* Mudd, *Verrucaria foveolata* (Flörke) A. Massal., *V. muralis*, *V. nigrescens* s.lat. and *V. viridula* (Schrad.) Ach.

##### Etymology.

The name refers to the flattened apothecia of the species.

##### Notes.

The species is characterised by plane to slightly convex apothecia. *Protoblastenia pseudocompressa* is a sister species with a rather similar morphology. However, *P. pseudocompressa* often has convex apothecia which have not been seen in *P. compressa*. The occurrence of *Protoblastenia pseudocompressa* has been confirmed only in the Kuusamo-Salla area ca. 800 kilometres north from the distribution area of *P. compressa*. *Protoblastenia incrustans* differs in having apothecia that leave pits (*P. compressa* rarely leaves shallow pits). *Protoblastenia coniasis* differs in having a yellow thallus that gradually turns brownish-rose in the herbarium (Poelt 1957). *Protoblastenia compressa* may also be difficult to separate from *P. rupestris*, even though they are not closely related. However, *P. compressa* usually has thinner apothecia and a thinner thallus which is rarely areolate (mostly areolate in *P. rupestris*). ITS may sometimes be needed for unambiguous identification of *P. compressa*.

##### Other sequenced specimens.

Finland • Varsinais-Suomi, Kemiönsaari (Västanfjärd), Södersundvik, Näsudden, calcareous rock outcrop on shore of the Baltic Sea, cliff, on N-facing wall, rather scarce, 12 m a.s.l., 60°03'N, 22°46'E, 25.8.2009, J. Pykälä 36521 (H); • Parainen (Korppoo), Elfsjö, Stora Limskär Island, abandoned lime quarry, beneath N-facing wall, bottom, on pebbles, 4 m a.s.l., 60°09'N, 21°26'E, 22.6.2010, J. Pykälä 38349 (H); • Parainen (Korppoo), Åfvensår, Ronudden, abandoned lime quarry, on NW-facing wall, 2 m a.s.l., 60°18'N, 21°32'E, 2.7.2010, J. Pykälä 38646 (H); • Parainen (Korppoo), Åfvensår, Ronudden, abandoned lime quarry on shore of the Baltic Sea, on overhanging NW-facing wall, 2 m a.s.l., 60°17'N, 21°32'E, 15.7.2010, J. Pykälä 38965 (H); • Salo (Suomusjärvi), Rautsuo, 200 m NE of Kukutin, herb-rich forest, abandoned lime quarry, on NW-facing wall, 85 m a.s.l., 60°21'N, 23°37'E, 28.9.2010, J. Pykälä 41376 (H); • Parainen, Skräbböle, Skräbböle lime quarry, by the lime quarry, N-slope, on stone, 24 m a.s.l., 60°17'N, 22°17'E, 5.10.2010, J. Pykälä 41511b (H); • Parainen, Attu, Skötudden SW, abandoned lime quarry on shore of the Baltic Sea, on NE-facing wall, 2 m a.s.l., 60°11'N, 22°18'E, 16.6.2011, J. Pykälä 42124 (H).

#### ﻿ Protoblastenia dolomitica

Taxon classificationFungiLecanoralesPsoraceae

Pykälä & Myllys sp. nov.

B5E2DF04-91C0-5E7B-94AC-65B8593D8E2E

859628

Fig. 2D

##### Diagnosis.

Differs from *P. siebenhaariana* in having more densely occurring apothecia and from *P. arupii* in having a rimose to areolate thallus and often olive-yellow apothecia.

##### Type.

Finland • Enontekiön Lappi, Enontekiö, Porojärvet, Toskalharji, Toska­ljärvi N, fell, brook, W-shore, dolomite rock outcrop, on N-slope, 710 m a.s.l., 69°11'N, 21°26'E, 3 August 2011, J. Pykälä 43466 (H9235018 – holotype, GenBank accession number: PV766671).

##### Description.

Thallus white to whitish-grey, rarely pale brown, rimose to areolate, areoles 0.2–0.9 mm, ca. 0.03–0.25 mm thick, K-, C-, UV-, one specimen UV+ bluish-white, algal cells 4–8(–12) μm. Apothecia yellow to dirty orange-yellow, sometimes olive-yellow, 0.15–0.7 mm, plane, slightly convex to convex, 1/2-immersed in thallus; ca. 40–160 apothecia / cm^2^. Epihymenium dirty olive-yellow to orange-brown, 12–20 μm thick, K+ violet. Hymenium 50–70 μm thick. Hypothecium dark brown, reddish-brown or usually violet, ca. 40–100 μm thick. Paraphyses ca. 1.5–2 μm thick, apex not thickened to slightly thickened, branching. Ascospores 0-septate, (7.6–)11.0–12.8–14.7(–19.4) × (4.4–)4.8–5.4–5.9(–7.4) μm (n = 117).

##### Habitat and distribution.

This species has been found only in the parishes of Salla (in the biogeographical Province Ks) and Enontekiö (biogeographical Province EnL) in N Finland. It grows on dolomite rock outcrops, once collected from dolomite stones and pebbles. The species may prefer sun-exposed and half-shady sites on fells and in gorges. Companion species include, for example, *Acarospora glaucocarpa*, *Hymenelia rhodopis* (Sommerf.) Lutzoni, *Opegrapha dolomitica* (Arnold) Clauzade & Cl. Roux ex Torrente & Egea, *Polyblastia* spp., *Protoblastenia fennoarctica*, *Synalissa ramulosa* (Bernh.) Körb., *Thelidium auruntii* (A. Massal.) Kremp. and *T. declivum* Pykälä & Myllys.

##### Etymology.

The species grows on dolomite rocks.

##### Notes.

*Protoblastenia dolomitica* is closely related to *P. westbergii*, which has slightly larger apothecia, broader spores and sometimes a thicker hypothecium. The specimens have been previously identified as *P. siebenhaariana* in Finland. However, this species has only 1–10 apothecia / cm^2^ (Kainz and Rambold 2004) and the Finnish specimens are not closely related to a GenBank specimen of *P. siebenhaariana*. *Protoblastenia dolomitica* is characterised by rather small, often dirty olive-yellow apothecia and a thin hypothecium. *Protoblastenia arupii*, based on the inspection of only one known specimen, has orange apothecia, on average, broader spores and an only slightly thinner rimose thallus. More material from the morphologically related species is needed to determine whether *P. dolomitica* can be identified by morphology only.

##### Other specimens examined.

Finland • Koillismaa, Salla, Oulanka National Park, Pikkuköngäs, bank of River Oulankajoki, high cliff, calciferous (dolomite) schistose rock, on overhanging SW-facing wall, 180 m a.s.l., 66°25'N, 29°09'E, 10.8.2009, J. Pykälä 36072 (H); • Salla, Oulanka National Park, Pikkuköngäs, bank of River Oulankajoki, high cliff, calciferous (dolomite) schistose rock outcrop, on SE-facing wall, 182 m a.s.l., 66°25'N, 29°08'E, 4.8.2010, J. Pykälä 39217 (H); • Salla, Oulanka National Park, Pikkuköngäs, bank of River Oulankajoki, high cliff, calciferous (dolomite) schistose rock outcrop, beneath SW-facing wall, on pebble, 180 m a.s.l., 66°25'N, 29°08'E, 13.8.2010, J. Pykälä 39851 (H); • Enontekiön Lappi, Enontekiö, Porojärvet, Toskalharji, Toskaljärvi N, fell, dolomite rock outcrop, gentle SE-slope, on dolomite pebbles, 730 m a.s.l., 69°12'N, 21°26'E, 2.8.2011, J. Pykälä 43340 (H); • Enontekiö, Porojärvet, Toskalharji, Toskaljärvi N, lake shore, dolomite scree, on dolomite stones, 705 m a.s.l., 69°11'N, 21°26'E, 7.8.2011, J. Pykälä 43855 (H); • Enontekiö, Kilpisjärvi, Saana, fell, steep NE-slope, dolomite rock outcrop, on NE-facing wall, 820 m a.s.l., 69°02'N, 20°51'E, 11.8.2011, J. Pykälä 44115 (H).

#### ﻿ Protoblastenia ekmanii

Taxon classificationFungiLecanoralesPsoraceae

Pykälä & Myllys sp. nov.

D4035663-27F6-5D71-B1AE-EBE6C97A9B99

859630

Fig. 3A

##### Diagnosis.

Differs from *P. borealis* in having a thicker hypothecium.

##### Type.

Finland • Varsinais-Suomi, Salo (Kisko), Kirkonkylä, 450 m NW of Kolko, shore of Määrjärvi Lake, calcareous rock outcrop, on W-slope, 43 m a.s.l., 60°12'N, 23°31'E, 20 May 2010, J. Pykälä 37508 (H9224071 – holotype, GenBank accession number: PV766674).

##### Description.

Thallus white to greyish-white, rimose to areolate, areoles 0.3–0.6 mm, ca. 0.05–0.2 mm thick, K-, UV+ bluish-white, algal cells 5–8 μm. Apothecia dirty brown-orange, 0.4–0.8 mm, convex to strongly convex, superficial; ca. 30–50 apothecia / cm^2^. Epihymenium dark orange-brown, 10–20 μm thick, K+ violet. Hymenium 50–70 μm thick. Hypothecium yellowish-white, ca. 120–200 μm thick, in some apothecia base patchily red-violet. Paraphyses ca. 2–2.5 μm thick, apex not or slightly thickened. Ascospores 0-septate, (8.1–)8.7–10.2–11.6(–12.5) × (5.0–)5.1–5.9–6.6(–7.2) (n = 12).

**Figure 3.**
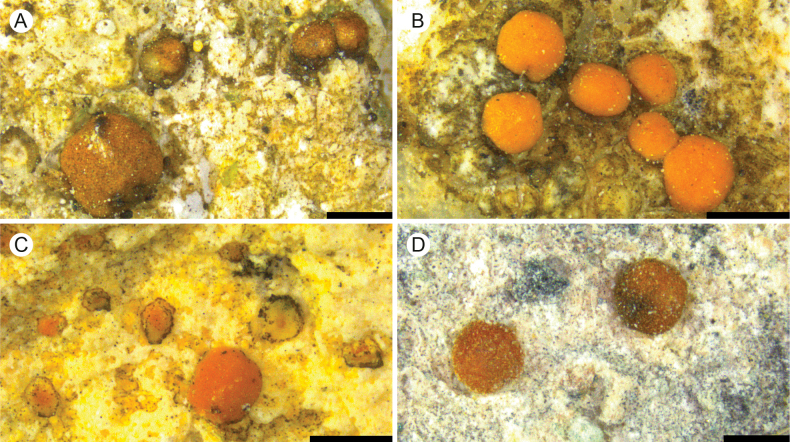
A. *Protoblastenia ekmanii* (holotype); B. *P. fennoarctica* (holotype); C. *P. minuta* (holotype); D. *P. oulankaensis* (holotype). Scale bars: 0.5 mm.

##### Habitat and distribution.

Only one specimen is known from a calcareous rock outcrop on a lake shore in SW Finland. The sun-exposed site suggests that the species may have a southern distribution. The species is apparently very rare in Finland. Companion species include *Acarospora glaucocarpa*, *Clauzadea monticola*, *Placynthium nigrum* (Huds.) Gray and *Thelidium incavatum*.

##### Etymology.

The name is in honour of Dr Stefan Ekman (Uppsala) for his many important contributions on the phylogeny and taxonomy of lichens in Fennoscandia and elsewhere.

##### Notes.

The species is closely related to *P. borealis* (only 2.5% difference in ITS), but seems to differ in having a thicker hypothecium. The hypothecium of *P. borealis* is very variable, but in no specimens does it reach the upper limit of the thickness in the *P. ekmanii* specimen. The Finnish specimens of *P. calvella* have orange-yellow to orange apothecia and thus resemble *P. ekmanii*. More material is needed to determine whether the species can be unambiguously morphologically distinguished from *P. calvella*.

#### ﻿ Protoblastenia fennoarctica

Taxon classificationFungiLecanoralesPsoraceae

Pykälä & Myllys sp. nov.

EC602A5E-4965-5BDD-8DC0-FBF1BFE0AD57

859631

Fig. 3B

##### Diagnosis.

Differs from *P. calvella* in having thinner hymenium, from *P. minuta* in having rimose to areolate thallus and from *P. rikkinenii* in usually having more densely occurring apothecia.

##### Type.

Finland • Enontekiön Lappi, Enontekiö, Porojärvet, Toskalharji, 1.2 km NE of Toskaljärvi, fell, SW-slope, scree, on dolomite boulder, 875 m a.s.l., 69°12'N, 21°28'E, 5 August 2011, J. Pykälä 43656 (H9220129 – holotype, GenBank accession number: PV766677).

##### Description.

Thallus white to pale greyish-brown, rimose to areolate, areoles 0.15–1.0 mm, ca. 0.03–0.25 mm thick, K-, C-, UV-, algal cells 5–10 μm. Apothecia yellow, orange-yellow to orange, 0.3–0.9 mm, plane to strongly convex, immersed in thallus to superficial; ca. 20–110 apothecia / cm^2^. Epihymenium orange-yellow to brown, 12–20 μm thick, K+ violet. Hymenium 50–80 μm thick. Hypothecium colourless to yellow, base sometimes patchily reddish-brown, ca. 60–280 μm thick. Paraphyses ca. 2–3 μm thick, cells 5–8 μm long, apex not thickened to slightly thickened, sparingly branched and anastomosing. Ascospores 0-septate, (6.6–)8.7–10.0–11.3(–12.5) × (4.2–)4.8–5.3–5.9(–7.2) μm (n = 93).

##### Habitat and distribution.

This species has been found only in the Parish Enontekiö (biogeographical Province EnL in NW Finland). It grows on fells on dolomite rock outcrops, boulders and pebbles. Companion species include *Enchylium polycarpon*, *Farnoldia jurana*, *Physcia caesia* (Hoffm.) Fürnr., *Polyblastia* spp., *Protoblastenia dolomitica* and *Verrucaria vacillans* Pykälä & Myllys.

##### Etymology.

The species occurs in the most northern, almost arctic (oroarctic) sites in Finland (Fennia).

##### Notes.

*Protoblastenia fennoarctica* morphologically resembles *P. calvella*, *P. minuta*, *P. remota* and *P. rikkinenii* in having a pale hypothecium and small spores. In the ITS phylogeny, the species is most closely related to *P. rikkinenii* (Fig. 1). *Protoblastenia rikkinenii* differs in having more sparsely occurring apothecia, but more material is needed to confirm whether the two species can be identified by morphology. *Protoblastenia calvella* has a thicker hymenium (80–100 μm thick). *Protoblastenia minuta* and *P. remota* have an endolithic to thin thallus and *P. minuta* has smaller spores compared to *P. fennoarctica*, while *P. remota* may have slightly larger spores.

##### Other specimens examined.

Finland • Enontekiön Lappi, Enontekiö, Porojärvet, Toskalharji, Toskalpahta fell, SW-slope, scree, on dolomite pebbles, 795 m a.s.l., 69°11'N, 21°29'E, 1.8.2011, J. Pykälä 43105 (H); • Enontekiö, Porojärvet, Toskalharji, Toskaljärvi N, fell, dolomite scree, gentle SE-slope, on dolomite boulder, 715 m a.s.l., 69°11'N, 21°26'E, 2.8.2011, J. Pykälä 43194 (H); • Enontekiö, Kilpisjärvi, Saana, fell, steep NE-slope, dolomite rock outcrop, on NE-facing wall, 820 m a.s.l., 69°02'N, 20°51'E, 11.8.2011, J. Pykälä 44116 (H).

#### ﻿ Protoblastenia lilacina

Taxon classificationFungiLecanoralesPsoraceae

Poelt & Vězda, Čas. slezsk. Mus. Opavě, Ser. A 19: 26 (1970)

26F7C1B6-4070-55EB-A01B-CB72E0DFB756

##### Description.

Thallus white to grey, endolithic, farinose to small areoles, K-, C-, UV- to UV+ bluish-white, algal cells 3–8 μm. Apothecia orange to dark orange, 0.2–0.6 mm, slightly convex to strongly convex, 1/4-immersed to superficial, sometimes leaving shallow pits, K- or K+ fairly weakly red; ca. 40–100 apothecia / cm^2^. Epihymenium (yellowish) orange-brown, 12–20 μm thick, K- to K+ orange or carmine red, not into solution. Hymenium 50–120 μm thick, with oil droplets. Hypothecium pale yellow, sometimes patchily violet, ca. 60–120 μm thick. Paraphyses ca. 2–3 μm thick, apex not thickened to slightly thickened, sparingly branched and anastomosing. Ascospores 0-septate, (8.4–)10.7–12.4–14.2(–16.6) × (5.2–)6.0–6.7–7.3(–7.8) μm (n = 30).

##### Habitat and distribution.

The species is rare and occurs only on calcareous rock outcrops and boulders in the hemi-boreal zone in SW Finland. This seems to be the northern limit of this predominantly temperate species. *Protoblastenia lilacina* grows both on fairly sun-exposed and shady habitats. It may prefer rather dry calcareous rocks. Companion species include, for example, *Bagliettoa baldensis* s.lat, *B. calciseda* (DC.) Gueidan & Cl. Roux, *Catillaria lenticularis* (Ach.) Th. Fr., *Hymenelia* spp., *Lecidella stigmatea* (Ach.) Hertel & Leuckert, *Staurothele rupifraga* (A. Massal.) Arnold, *Verrucaria deversa* Vain., *V. foveolata* and *V. viridula*.

##### Notes.

The species differs from most species of *Protoblastenia* in having K- or weakly K+ red apothecia. The closely-related *Protoblastenia oulankaensis* is the only other species with this characteristic. However, the two species have different distribution areas as *Protoblastenia oulankaensis* is only known from the Oulanka area in NE Finland. Morphologically, *P. oulankaensis* differs in having a thicker hypothecium and often more sparsely occurring apothecia (in most specimens 10–30 apothecia / cm^2^).

**Specimens examined**. Finland • Varsinais-Suomi, Kemiönsaari (Västanfjärd), Vesterillo, Strömmen 200–400 m SE, *Pinus sylvestris*-dominated forest, calcareous rock outcrop, on 70 cm high E-facing wall, 17 m a.s.l., 60°00'N, 22°44'E, 9.9.2009, J. Pykälä 37012 (H); • Lohja, Moisio, between Lohjanharjuntie and Nummentie roads, NW-slope of Lohjanharju esker, *Pinus sylvestris*-heath forest, on calcareous boulder, 82 m a.s.l., 60°15'N, 24°06'E, 18.6.2005, J. Pykälä 27009 H); • Lohja, Lohja, Pitkäniemi industrial area, 5 m from shore of Lohjanjärvi Lake, deciduous forest, calcareous rock outcrop, on NW-facing 2 m high rock wall, 35 m a.s.l., 60°15'N, 24°02'E, 19.8.2005, J. Pykälä 27629 (H); • Lohja, Lohja, Pitkäniemi industrial area, deciduous forest ca. 5 m from shore of Lohjanjärvi Lake, calcareous rock outcrop, on 1.7 m high overhanging NW-facing wall, 35 m a.s.l., 60°15'N, 24°02'E, 12.7.2021, J. Pykälä 57976 (H).

#### ﻿ Protoblastenia minuta

Taxon classificationFungiLecanoralesPsoraceae

Pykälä & Myllys sp. nov.

A4AF1B5F-60E4-5D47-BAD7-F03E6789169D

859654

Fig. 3C

##### Diagnosis.

Differs from the other *Protoblastenia* species with a pale hypothecium in having small apothecia and spores and apothecia which leave pits.

##### Type.

Finland • Enontekiön Lappi, Enontekiö, Kilpisjärvi, Saana Nature Reserve, E-part, fell, steep SW-slope, dolomite rock outcrop, on S-facing wall, scarce, 715 m a.s.l, 69°02'N, 20°51'E, 12 August 2011, J. Pykälä 44275 (H9250941 – holotype, GenBank accession number: PV766683).

##### Description.

Thallus ochraceous, endolithic, K-, UV-. Apothecia orange, 0.2–0.4 mm, slightly convex to strongly convex, immersed in rock to superficial, leaving shallow to fairly deep pits; ca. 80 apothecia / cm^2^. Epihymenium brown-yellow, 10–12 μm thick, K+ violet. Hymenium 45–55 μm thick. Hypothecium colourless to yellow, ca. 70–100 μm thick. Paraphyses ca. 2–3 μm thick, apex not thickened to slightly thickened. Ascospores 0-septate, (7.3–)7.6–7.9–8.3(–8.8) × (4.1–)4.3–4.5–4.7(–4.8) μm (n = 9).

##### Habitat and distribution.

Only one specimen has been found on the calcareous Saana fell in NE Finland, on a dolomite rock outcrop. Companion species include *Acarospora macrospora* (Hepp) A. Massal. ex Bagl., *Enchylium polycarpon*, *Placynthium asperellum* (Ach.) Trevis. s.lat, *Romjularia lurida* (Ach.) Timdal and *Thalloidima alutaceum* Anzi.

##### Etymology.

The name refers to the small size of the apothecia and spores.

##### Notes.

The species is rather isolated amongst the sequenced species of *Protoblastenia* (minimum difference from other species over 9% in ITS). The species has smaller apothecia and spores than most other *Protoblastenia* species with a pale hypothecium. Furthermore, other Finnish *Protoblastenia* species do not leave pits or only rarely leave shallow pits. However, only one specimen is known and the species may be expected to be morphologically more variable. More material is needed to evaluate whether the species can be identified by morphology only. *Protoblastenia saanaensis* has a rimose thallus and the apothecia do not leave pits. *Protoblastenia szaferi* differs in having larger apothecia and spores (see Hafellner (2006)).

#### ﻿ Protoblastenia oulankaensis

Taxon classificationFungiLecanoralesPsoraceae

Pykälä & Myllys sp. nov.

FE5B4688-BA79-556A-B554-01286B667578

859655

Fig. 3D

##### Diagnosis.

Differs from *P. lilacina* in often having slightly larger and more sparse apothecia and an often thicker hypothecium.

##### Type.

Finland • Koillismaa, Salla, Oulanka National Park, W of Savikoski, cliff, dolomite rock outcrop, on NE-facing wall, 190 m a.s.l., 66°25'N, 29°10'E, 17 August 2010, J. Pykälä 40201 (H9224130 – holotype, UPS – isotype, GenBank accession number: PV766686).

##### Description.

Thallus white to whitish-grey, endolithic, farinose or rimose, ca. 0–0.1 mm thick, K-, C-, UV- to UV+ bluish-white, algal cells 5–9 μm. Apothecia orange to dark orange, 0.3–0.9 mm, convex to strongly convex, 1/4-immersed to superficial, often leaving shallow pits, K+ weakly red; ca. 10–50 apothecia / cm^2^ (in one specimen, ca. 50–80 apothecia / cm^2^). Epihymenium dark orange-brown, 12–25 μm thick, K- to K+ orange or carmine red. Hymenium 70–120 μm thick, with oil droplets, in one specimen, partly violet in few apothecia. Hypothecium colourless to yellow, more rarely patchily red-brown, ca. 60–220 μm thick. Paraphyses ca. 2–3 μm thick, apex not thickened to slightly thickened, sparingly branched and anastomosing. Ascospores 0-septate, (8.8–)11.8–13.9–16.0(–18.8) × (5.2–)6.6–7.4–8.2(–9.6) μm (n = 92).

##### Habitat and distribution.

The species occurs only in the Oulanka area in the parishes of Kuusamo and Salla in NE Finland. It grows on N- and S-facing walls of dolomite rock outcrops. Companion species include, for example, *Diploschistes gypsaceus* (Ach.) Zahlbr., *Gyalecta jenensis* (Batsch) Zahlbr., *Lempholemma isidioides* (Nyl. ex Arnold) H. Magn., *Protoblastenia borealis*, *Sagiolechia protuberans*, *Scytinium parvum*, *Verrucaria foveolata* and *Xanthocarpia crenulatella* (Nyl.) Frödén, Arup & Søchting s.lat. Based on the ITS phylogeny, one GenBank specimen of *P. lilacina* from northern Italy (AY425629) belongs to *P. oulankaensis* (Fig. 1).

**Etymology**. In Finland, the species occurs only in the Oulanka area in the northeast.

##### Notes.

*Protoblastenia oulankaensis* is closely related to *P. lilacina*, but differs clearly from that species in its ITS profile (10% difference). It is also morphologically rather similar to *P. lilacina*, having a weak K+ reaction of apothecia and large spores. *Protoblastenia oulankaensis* tends to have slightly larger apothecia, which usually occur more sparsely (in most specimens ca. 10–50 apothecia /cm^2^) and, on average, a thicker hypothecium. *Protoblastenia lilacina* is only known from the hemi-boreal zone of SW Finland, ca. 800 kilometres south to south-west.

##### Other specimens examined.

Finland • Koillismaa, Salla, Oulanka National Park, Savikoski 300 m N, *Pinus sylvestris*-forest, steep N-slope, dolomite rock outcrop, on NW-facing wall, st pc – sp, 180 m a.s.l., 66°25'N, 29°10'E, 10.8.2010, J. Pykälä 39629 (H); • Salla, Oulanka National Park, Savilamminniemi, shore of Savilampi Lake, cliff, dolomite rock outcrop, on E-facing wall, sp, 180 m a.s.l., 66°25'N, 29°10'E, 12.8.2010, J. Pykälä 39810 (H); • Salla, Oulanka National Park, Savilampi 1.4 km NE, bank of Savinajoki river, cliff, dolomite rock outcrop, upper slope, on NE-facing wall, 192 m a.s.l., 66°26'N, 29°11'E, 23.8.2011, J. Pykälä 44928 (H); • Salla, Hautajärvi, Kurtinniittykuru, dolomite rock outcrop, on SW-facing wall, 195 m a.s.l., 66°26'N, 29°09'E, 24.8.2011, J. Pykälä 44949 (H); • Salla, Hautajärvi, Kurtinniittykuru, dolomite rock outcrop, on SE-facing wall, 195 m a.s.l., 66°26'N, 29°09'E, 24.8.2011, J. Pykälä 44953 (H); • Kuusamo, Juuma, Myllyniemi, dolomite rock outcrop, on NE-facing wall, 235 m a.s.l., 66°16'N, 29°22'E, 11.8.2020, J. Pykälä 55995 (H).

#### ﻿ Protoblastenia pseudocompressa

Taxon classificationFungiLecanoralesPsoraceae

Pykälä & Myllys sp. nov.

2B50F168-D1BC-5381-A71F-8B819B54D36C

859656

Fig. 4A

##### Diagnosis.

Differs from *P. compressa* in often having convex apothecia.

##### Type.

Finland • Koillismaa, Salla, Onkamo, Latvarova SW, clear-cut heath forest, on calcareous boulder, 258 m a.s.l., 66°42'N, 28°055'E, 9 August 2021, J. Pykälä 58457 (H9250942 – holotype, UPS – isotype, GenBank accession number: PV766696).

##### Description.

Thallus white to grey, continuous, rimose (usually) to areolate, rarely endolithic, ca. 0–0.15 mm thick, K-, C-, UV- to UV+ bluish-white, algal cells 6–12 μm. Apothecia orange-yellow to orange, 0.2–0.7 mm, plane to strongly convex, 1/4–1-immersed in thallus or rock to superficial, sometimes leaving shallow pits; ca. 20–120 apothecia / cm^2^. Epihymenium yellow-brown to orange-brown, 12–18 μm thick, K+ violet. Hymenium 50–60 μm thick. Hypothecium colourless to pale yellow, rarely pink (in one specimen), ca. 50–120 μm thick. Paraphyses ca. 1.5–2.5 μm thick, sparsely branched and anastomosing. Ascospores 0-septate, (6.8–)9.1–10.6–12.1(–13.0) × (4.2–)4.7–5.5–6.2(–8.0) μm (n = 51).

**Figure 4.**
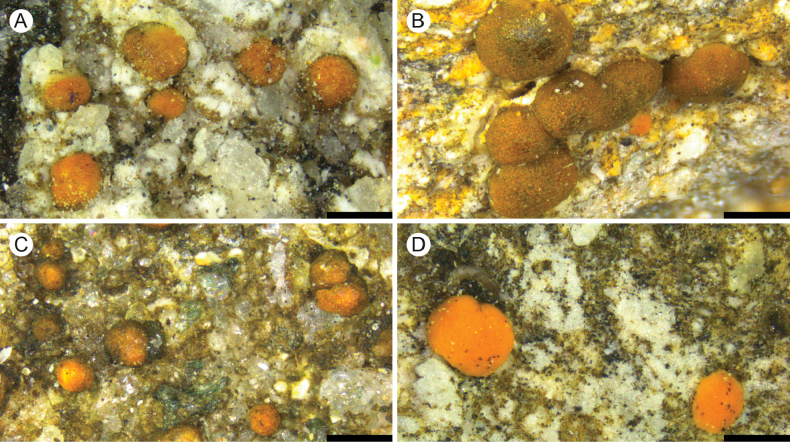
A. *Protoblastenia pseudocompressa* (holotype); B. *P. pseudoterricola* (holotype); C. *P. remota* (holotype); D. *P. rikkinenii* (holotype). Scale bars: 0.5 mm.

##### Habitat and distribution.

*Protoblastenia pseudocompressa* has been verified by ITS only from the Oulanka area (parishes Kuusamo and Salla) in NE Finland. However, a morphologically similar specimen was found in the neighbouring biogeographical Province of Kainuu. The species cannot be identified with certainty by morphology only and it is likely that more populations occur in the Oulanka area. It mainly grows on dolomite rock outcrops (one specimen was collected from a dolomite boulder) and may prefer half-shady habitats. Typical companion species are *Acarospora glaucocarpa*, *Clauzadea monticola*, *Farnoldia jurana*, *Hymenelia prevostii* (Duby) Kremp., *H. rhodopis*, *Lathagrium fuscovirens*, *Placynthium nigrum*, *Thelidium declivum*, *Verrucaria deversa*, *V. muralis*, *V. kuusamoensis* Pykälä, Kantelinen & Myllys and *V. subtilis*.

##### Etymology.

The name refers to the close relationship in ITS and morphological similarity to *P. compressa*.

##### Notes.

*Protoblastenia pseudocompressa* is closely related to *P. compressa* and the two species are also morphologically similar. *Protoblastenia pseudocompressa* has, on average, slightly smaller spores than *P. compressa*, but there is a wide overlap. Furthermore, *P. pseudocompressa* specimens with plane to slightly convex apothecia may be confused with *P. compressa*. However, the apothecia of *P. pseudocompressa* are often convex, while the apothecia of *P. compressa* are always plane to slightly convex. Based on the distribution of sequenced specimens, the two species are geographically well separated. *Protoblastenia pseudocompressa* specimens with convex apothecia may also be confused with *P. borealis*, which, however, often has a thicker hymenium.

##### Other specimens examined.

Finland • Koillismaa, Kuusamo, Oulanka National Park, Pikkukönkäänkuru, dolomite rock outcrop, small W-facing wall, 175 m a.s.l., 66°21'N, 29°19'E, 8.8.2009, J. Pykälä 35901 (H); • Salla, Oulanka National Park, 400 m N of Savilampi, bank of River Savinajoki, dolomite rock outcrop, on NW-facing wall, 178 m a.s.l., 66°25'N, 29°10'E, 13.8.2009, J. Pykälä 36325 (H); • Kuusamo, Oulanka National Park, Kiutaköngäs 400 m N, SW-slope, *Pinus sylvestris*-dominated forest, small dolomite rock outcrop, on SW-facing wall, scarce, 175 m a.s.l., 66°22'N, 29°19'E, 6.8.2010, J. Pykälä 39367 (H); • Kuusamo, Oulanka National Park, Kiutaköngäs, bank of Oulankajoki River, by the rapids, calciferous (dolomite) schistose rock outcrop, on rather flat rock, 150 m a.s.l., 66°22'N, 29°20'E, 10.8.2010, J. Pykälä 39693 (H); • Kuusamo, Oulanka, Putaanoja, 500 m W-NW of Hautala, dolomite rock outcrop, on E-facing wall, scarce, 232 m a.s.l., 66°22'N, 29°25'E, 15.8.2010, J. Pykälä 39987 (H).

#### ﻿ Protoblastenia pseudoterricola

Taxon classificationFungiLecanoralesPsoraceae

Pykälä & Myllys sp. nov.

7265A0B0-1882-5346-AC78-143B76781C9F

859657

Fig. 4B

##### Diagnosis.

Differs from *P. terricola* in having a thinner thallus and hypothecium.

##### Type.

Finland • Enontekiön Lappi, Enontekiö, Kilpisjärvi, Saana, fell, steep NE-slope, dolomite rock outcrop, beneath NE-facing wall, on dolomite pebbles, 820 m a.s.l., 69°02'N, 20°51'E, 11 August 2011, J. Pykälä 44157 (H9235068 – holotype, GenBank accession number: PV766697).

##### Description.

Thallus white, rimose, ca. 0.05–0.1 mm thick, K-, UV+ bluish-white. Apothecia orange (young apothecia) to dirty orange-brown, 0.3–0.8 mm, convex to strongly convex, superficial; ca. 30 apothecia / cm^2^. Epihymenium orange-brown, 10–15 μm thick, K+ violet. Hymenium 70 μm thick. Hypothecium dark reddish-brown to violet, ca. 60–100 μm thick. Ascospores 0-septate, (7.9–)8.6–9.9–11.2(–12.4) × (4.5–)4.8–5.5–6.2(–7.0) μm (n = 20).

##### Habitat and distribution.

Only one specimen of this species is known. It was collected from dolomite pebbles beneath an NE-facing wall of a dolomite rock outcrop on the Saana fell (NW Finland). Companion species include *Farnoldia jurana*, *Polyblastia inconspicua* Savić & Tibell and *Thelidium huuskonenii* Pykälä & Myllys.

##### Etymology.

The name refers to the similarities in morphology and ITS to *P. terricola*.

##### Notes.

The species is closely related to *P. terricola* and *P. violacea*. *Protoblastenia terricola* has rimose to areolate thallus (when growing on rock) mainly exceeding 0.1 mm in thickness and an often thicker hypothecium. *Protoblastenia violacea* has orange apothecia and smaller spores. More material is needed to confirm whether these three species can be distinguished by morphology only. *Protoblastenia westbergii* resembles the new species, but has more densely occurring apothecia (ca. 40–160 apothecia / cm^2^) and larger spores.

#### ﻿ Protoblastenia remota

Taxon classificationFungiLecanoralesPsoraceae

Pykälä & Myllys sp. nov.

BDE342D3-B7B8-547D-84F9-CF3E67DE85D5

859658

Fig. 4C

##### Diagnosis.

Differs from *P. borealis* and *P. pseudocompressa* by often smaller more convex apothecia and a smaller thallus.

##### Type.

Finland • Varsinais-Suomi, Perniö, Lupaja, Alhonmäki, abandoned lime quarry, on W-slope, 9 August 1991, J. Pykälä 8547 (H9250943 – holotype, GenBank accession number: PV766698).

##### Description.

Thallus white to grey, endolithic to sparse small areoles, 0.2–0.5 mm wide, ca. 0–0.15 mm thick, K-, C-, UV- to UV+ bluish-white. Apothecia orange, 0.2–0.6 mm, convex to strongly convex, 1/2-immersed to mainly superficial; ca. 15–80 apothecia / cm^2^. Epihymenium yellow-brown to dark orange, 12–20 μm thick, K+ violet. Hymenium 50–80 μm thick. Hypothecium colourless to pale yellow, ca. 100–200 μm thick. Paraphyses ca. 2–2.5 μm thick, apex not thickened to thickened to ca. 3–5 μm thick. Ascospores 0-septate, (8.4–)9.8–11.4–12.9(–14.4) × (4.8–)5.0–5.6–6.1(–7.1) μm (n = 36).

##### Habitat and distribution.

Two specimens of this species are known: one from a lime quarry in SW Fnland and one from a calciferous schistose cliff in NE Finland. Companion species include *Lathagrium fuscovirens*, *Mycobilimbia tetramera* (De Not.) Vitik., Ahti, Kuusinen, Lommi & T. Ulvinen ex Hafellner & Türk and *Verrucaria deversa*. Furthermore, two closely-related specimens from North America and previously identified as Protoblastenia aff. rupestris (MZ922194, MZ922195) (Munger et al. 2022) may belong to *P. remota* (Fig. 1).

##### Etymology.

The name refers to the large difference in ITS of the species compared to other *Protoblastenia* species (differs by ca. 15–18% from other species).

##### Notes.

Based on ITS sequences, this species is not closely related to any sequenced *Protoblastenia* species. The species is morphologically a typical *Protoblastenia* and very difficult to distinguish from several species, particularly from *P. borealis* and *P. pseudocompressa*. However, these two species have, on average, less convex apothecia, a thinner hypothecium and often a better developed thallus, but there is a wide overlap. In addition, *Protoblastenia borealis* often has larger apothecia (up to 1 mm).

##### Other specimens examined.

Finland • Koillismaa, Kuusamo, Oulanka National Park, Päähkänänkallio, high cliff, calciferous (dolomite) schistose rock outcrop, on SE-facing wall, st pc, 195 m a.s.l., 66°16'N, 29°31'E, 7.8.2010, J. Pykälä 39455 (H).

#### ﻿ Protoblastenia rikkinenii

Taxon classificationFungiLecanoralesPsoraceae

Pykälä & Myllys sp. nov.

3E736102-4570-5DF6-B9E3-35C87959A1E5

859659

Fig. 4D

##### Diagnosis.

Differs from *P. fennoarctica* in always having convex to strongly convex apothecia and from *P. saanaensis* and *P. timdalii* in having sparsely occurring apothecia and a thick hypothecium.

##### Type.

Finland • Enontekiön Lappi, Enontekiö, Porojärvet, Toskalharji, Toska­ljärvi N, fell, dolomite rock outcrop, on dolomite pebbles, 735 m a.s.l., 69°12'N, 21°26'E, 2 August 2011, J. Pykälä 43252 (H9250944 – holotype, GenBank accession number: PV766700).

##### Description.

Thallus white, grey to pale brown, rimose to areolate, ca. 0.05–0.2 mm thick, K-, C-, UV-, algal cells 5–9 μm. Apothecia yellow-orange, 0.3–0.8 mm, convex to strongly convex, 1/2-immersed to superficial; ca. 20–40 apothecia /cm^2^. Epihymenium dirty yellow to orange-yellow, 12–18 μm thick, K+ violet. Hymenium 50–70 μm thick. Hypothecium pale yellow, ca. 80–300 μm thick. Paraphyses ca. 2–2.5 μm thick, apex slightly thickened to 3 μm thick. Ascospores 0-septate, (7.4–)8.5–9.6–10.7(–12.6) × (4.4–)4.7–5.1–5.5(–6.3) μm (n = 52).

##### Habitat and distribution.

The species occurs on dolomite rock outcrops, dolomite stone and dolomite pebbles on the fells. It may prefer sun-exposed sites. Companion species include *Halecania alpivaga* (Th. Fr.) M. Mayrhofer, *Hymenelia heteromorpha* (Kremp.) Lutzoni, *H. prevostii*, *H. rhodopis*, *Lecidea polycocca* Sommerf., *Polyblastia* sp., *Rhizocarpon petraeum*, *Thelidium huuskonenii* and *Verrucaria lapponica* Pykälä. One GenBank sequence (KY266981) originates from northern Norway.

##### Etymology.

The name is in honour of Prof Jouko Rikkinen (Helsinki) for his innovative work on various fields of lichenology.

##### Notes.

The species is related to *P. fennoarctica*, which often has more densely occurring, often plane and/or yellow apothecia. However, an ITS sequence may be needed for unambiguous identification of specimens. *Protoblastenia saanaensis* and *P. timdalii* have a thinner hypothecium and more densely occurring apothecia. *Protoblastenia calvella* has more densely occurring apothecia and slightly larger spores.

##### Other specimens examined.

Finland • Enontekiön Lappi, Enontekiö, Kilpisjärvi, Saana Nature Reserve, W-part, fell, dolomite rock outcrop, on W-facing wall, 700 m a.s.l., 69°03'N, 20°48'E, 9.8.2011, J. Pykälä 43910 (H); • Enontekiö, Kilpisjärvi, Saana Nature Reserve, fell, steep SW-slope, dolomite rock outcrop, beneath SW-facing wall, on dolomite stone, 840 m a.s.l., 69°02'N, 20°50'E, 14.8.2011, J. Pykälä 44387 (H).

#### ﻿ Protoblastenia rupestris

Taxon classificationFungiLecanoralesPsoraceae

(Scop.) J. Steiner, Verh. Kaiserl.-Königl. zool.-bot. Ges. Wien 61: 47 (1911)

CE2CF32D-1453-527D-AFB5-185219F1D87F

Lichen rupestris Scop., Fl. carniol., Edn 2 (Wien) 2: 363 (1772).

##### Description.

Thallus whitish-grey to rarely pale brown, rimose to predominantly areolate, areoles 0.1–0.8 mm, ca. 0.1–0.2 mm thick, K-, C-, UV- to UV+ bluish-white, algal cells 4–12 μm. Apothecia yellow-orange to orange, 0.2–0.9 mm, slightly convex to convex, 3/4-immersed in thallus to superficial; ca. 40–100 apothecia / cm^2^. Epihymenium orange-brown, 12–20 μm thick, K+ violet. Hymenium 50–80 μm thick. Hypothecium pale yellow, ca. 60–80 μm thick. Paraphyses ca. 1.5–2.5 μm thick, apex not thickened to slightly thickened to 2–3 μm thick, sparingly branched and anastomosing. Ascospores 0-septate, (8.2–)9.1–10.3–11.6(–13.0) × (5.2–)5.6–6.3–7.1(–7.6) μm (n = 43).

##### Habitat and distribution.

Sequenced specimens were collected from rather sun-exposed calcareous rock outcrops in the hemi-boreal zone in Finland. The species may prefer calcareous rock outcrops close to shores.

##### Notes.

*Protoblastenia rupestris* sequences available in GenBank are heterogeneous and distributed in three different groups in our ITS phylogeny (Fig. 1). Specimens similar to those in Kainz and Rambold (2004) are likely to belong to *P. rupestris* s. stricto as the species was described from Central Europe. The sequence MZ159571 (UK) is related to *P. incrustans* and the sequence KY266981 (Norway) belongs in *P. rikkinenii*. Morphologically, the Finnish specimens of *P. rupestris* may be confused with the closely-related *P. borealis*. However, the *P. rupestris* specimens have a predominantly areolate thallus, while the thalli of *P. borealis* are more often rimose and usually do not exceed 0.1 mm in thickness. Furthermore, *P. borealis* often has a thicker hypothecium. Despite these minor differences, the two species often cannot be distinguished with certainty by morphology only.

##### Specimens examined.

Finland • Varsinais-Suomi, Lohja, Skraatila, Kirkko­vuori E, cliff, calcareous rock outcrop, on S-slope, 8.8.1990, J. Pykälä 5963 (H); • Lohja, Lohja, Pitkäniemi industrial area, by the sauna, on shore of Lohjanjärvi Lake, flat calcareous rock outcrop, 33 m a.s.l., 60°15'N, 24°02'E, 19.8.2005, J. Pykälä 27569 (H); • Karjalohja, Saarenpää, Saarenpäänniemi, calcareous rock outcrop on shore of Lohjanjärvi Lake, on small SW-facing wall, 35 m a.s.l., 60°13'N, 23°47'E, 7.10.2008, J. Pykälä 34043 (H); • Salo (Särkisalo), Kaukosalo, Klintinmäki, cliff, calciferous schistose rock outcrop, on SW-facing wall, st pc, 35 m a.s.l., 60°06'N, 22°58'E, 4.10.2011, J. Pykälä 46413 (H).

#### ﻿ Protoblastenia saanaensis

Taxon classificationFungiLecanoralesPsoraceae

Pykälä & Myllys sp. nov.

3817FD07-D221-5FE9-B64B-2ABC5DC32B13

859660

Fig. 5A

##### Diagnosis.

Differs from *P. minuta* in apothecia not leaving pits, from *P. rikkinenii* in having more densely occurring apothecia, from *P. fennoarctica* in having a thinner hypothecium and from *P. timdalii* in often having smaller apothecia.

##### Type.

Finland • Enontekiön Lappi, Enontekiö, Kilpisjärvi, Saana Nature Reserve, fell, steep SW-slope, dolomite rock outcrop, SW-slope, on dolomite pebbles, 835 m a.s.l., 69°02'N, 20°50'E, 14 August 2011, J. Pykälä 44431 (H9250945 – holotype, GenBank accession number: PV766707).

##### Description.

Thallus white, grey or pale brown, rimose to areolate, areoles 0.3–0.8 mm, ca. 0.03–0.1 mm thick, K-, C-, UV-, algal cells 5–12 μm. Apothecia yellow to orange-yellow, 0.3–0.5 mm, slightly convex to strongly convex, 3/4-immersed to superficial; ca. 20–80 apothecia / cm^2^. Epihymenium orange-yellow, 12–17 μm thick, K+ violet. Hymenium 50–70 μm thick. Hypothecium pale yellow (some orange-yellow in squash), ca. 40–100 μm thick. Paraphyses ca. 2–2.5 μm thick, apex not thickened to slightly thickened, branching. Ascospores 0-septate, (5.4–)7.1–8.4–9.7(–11.4) × (3.8–)4.3–4.8–5.4(–6.1) μm (n = 47).

**Figure 5.**
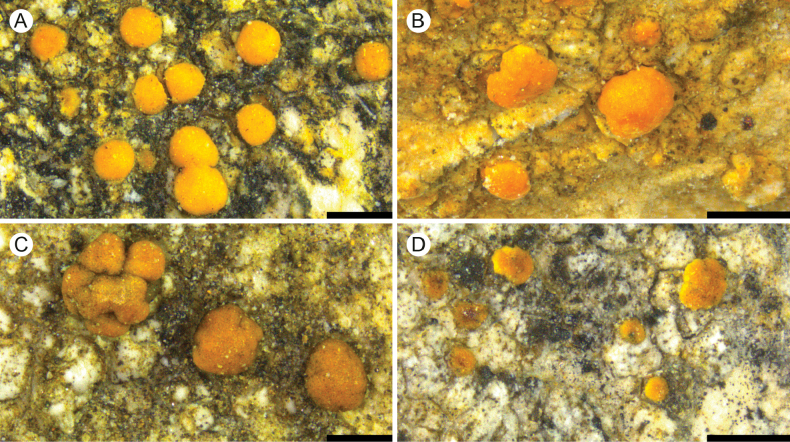
A. *Protoblastenia saanaensis* (holotype); B. *P. timdalii* (holotype); C. *P. violacea* (holotype); D. *P. westbergii* (holotype). Scale bars: 0.5 mm.

##### Habitat and distribution.

The species has only been found on the calcareous Saana fell in NW Finland. The specimens are from a dolomite stone and dolomite pebbles in sun-exposed to rather shady habitats. Companion species include *Acarospora glaucocarpa*.

##### Etymology.

The name refers to the Saana fell where both known specimens were collected.

##### Notes.

The species is most closely related to *P. timdalii* (ca. 5–6% difference in ITS) from which it is difficult to distinguish by morphology. It may have slightly smaller apothecia and spores. It also resembles *P. minuta* and the closely-related *P. rikkinenii* in having similar small spores. The only known specimen of *P. minuta* has apothecia which leave shallow to deep pits. *Protoblastenia rikkinenii* has sparsely occurring apothecia. *Protoblastenia fennoarctica* resembles *P. saanaensis*, but usually has a thicker hypothecium, slightly larger spores and sometimes plane apothecia.

##### Other specimens examined.

Finland • Enontekiön Lappi, Enontekiö, Kilpisjärvi, Saana Nature Reserve, fell, steep SW-slope, dolomite rock outcrop, under overhanging SW-facing wall, on dolomite stone, 835 m a.s.l., 69°02'N, 20°50'E, 14.8.2011, J. Pykälä 44438 (H).

#### ﻿ Protoblastenia terricola

Taxon classificationFungiLecanoralesPsoraceae

(Anzi) Lynge, Rep. Sci. Res. Norweg. Exped. Novaya Zemlya, 1921 43: 216 (1928)

9F8D5325-82E8-510C-8195-64D63E148805

Biatora rupestris var. terricola Anzi, Cat. Lich. Sondr.: 78 (1860).

##### Description.

Thallus white to whitish-grey, rimose to areolate, areoles 0.2–0.8 mm, ca. 0.05–0.2 mm thick, K-, C-, UV+ bluish-white. Apothecia dirty orange to brown-orange (some with an olive tinge), 0.4–0.9 mm, strongly convex, superficial, K+ violet; ca. 70–120 apothecia /cm^2^. Epihymenium dark orange-brown, 8–15 μm thick, K+ violet. Hymenium 60–100 μm thick. Hypothecium violet to violet-brown, 70–180 μm thick. Paraphyses ca. 2–2.5 μm thick, apex not thickened to slightly thickened, sparingly branched and anastomosing. Ascospores 0-septate, (9.4–)9.7–10.8–11.9(–12.4) × (4.7–)4.9–5.5–6.2(–7.3) μm (n = 16).

##### Habitat and distribution.

The species occurs only in northern Finland (biogeographical Provinces Ks and EnL) with one separate population in the biogeographical Province PK (not sequenced) in eastern Finland. It grows on calcareous rock outcrops and on calcareous soil.

##### Notes.

The specimens on calcareous soil (not sequenced) have a squamulose thallus, thus differing from the other species of *Protoblastenia* (Kainz and Rambold 2004). However, on dolomite rock outcrops, the thallus is more weakly developed, rimose to areolate. The apothecia of *P. terricola* tend to be dirty orange to brown. *Protoblastenia terricola* may be most difficult to distinguish from *P. pseudoterricola*. The only known specimen of *P. pseudoterricola* has a 60–100 μm thick hypothecium and a 0.05–0.1 mm thick thallus.

##### Specimen sequenced.

Finland • Koillismaa, Salla, Oulanka National Park, Savilampi 1.2 km NE, bank of Savinajoki River, dolomite rock outcrop, on SE-facing wall, 66°26'N, 29°11'E, 23.8.2011, J. Pykälä 44876 (H).

#### ﻿ Protoblastenia timdalii

Taxon classificationFungiLecanoralesPsoraceae

Pykälä & Myllys sp. nov.

9DCFD2E0-5AC4-5FAB-B581-918161BBE60C

859661

Fig. 5B

##### Diagnosis.

Differs from *P. saanaensis* in having slightly larger apothecia and spores.

##### Type.

Finland • Enontekiön Lappi, Enontekiö, Kilpisjärvi, Saana, fell, steep NE-slope, dolomite rock outcrop, on NE-facing wall, 820 m a.s.l., 69°02'N, 20°51'E, 11 August 2011, J. Pykälä 44177 (H9250946 – holotype, O – isotype, GenBank accession number: PV766711).

##### Description.

Thallus white, grey or pale brown, endolithic, rimose to areolate, areoles 0.1–0.4 mm, ca. 0–0.15 mm thick, K-, C-, UV-. Apothecia yellow-orange, 0.3–0.8 mm, convex to strongly convex, semi-immersed to superficial; ca. 40–60 apothecia / cm^2^. Epihymenium brown-yellow to dirty yellow, K+ violet. Hymenium 60–70 μm thick, pale, partly yellow. Hypothecium colourless to yellow, ca. 60–100 μm thick. Paraphyses ca. 2–2.5 μm thick, apex not thickened to slightly thickened. Ascospores 0-septate, (6.8–)7.9–9.2–10.5(–12.6) × (3.8–)4.2–4.8–5.5(–7.0) μm (n = 46).

##### Habitat and distribution.

Two specimens are known from the calcareous fells of Enontekiö, one from a NE-facing wall of a dolomite rock outcrop and one from dolomite stones on *Dryas octopetala* heath. This suggests that the species may have considerable microhabitat variation on the fells. Companion species include *Halecania alpivaga*, *Polyblastia* spp. and *Thelidium auruntii*.

##### Etymology.

The name is in honour of Prof Einar Timdal (Oslo) for his numerous important contributions to the taxonomy, biogeography and conservation of Fennoscandian lichens, including studies on the related genus *Psora* and an important technical edition of the Nordic lichen journal Graphis Scripta.

##### Notes.

The species is most closely related to *P. pseudoincrustans* ined. This undescribed species differs in having plane to convex apothecia, a thick hymenium (80–115 μm thick) and larger spores (mean 12 × 7 μm) (Kainz and Rambold 2004). However, in our phylogeny, *P. pseudoincrustans* is divided into two species. The closely-related *P. saanaensis* (differing ca. 5% in ITS) is also morphologically very similar to *P. timdalii*. *Protoblastenia saanaensis* may have slightly smaller apothecia and spores, but more material is needed to confirm this.

##### Other specimens examined.

Finland • Enontekiön Lappi, Enontekiö, Porojärvet, Toskalharji, 1.2 km NE of Toskaljärvi, fell, SW-slope, gentle E-slope, *Dryas* heath, on dolomite stones, 875 m a.s.l., 69°12'N, 21°28'E, 5.8.2011 J. Pykälä 43624 (H).

#### ﻿ Protoblastenia violacea

Taxon classificationFungiLecanoralesPsoraceae

Pykälä & Myllys sp. nov.

33C4E593-2077-503C-B3F1-9712B1F22336

859662

Fig. 5C

##### Diagnosis.

Differs from most *Protoblastenia* species with violet hypothecium in having a thick hypothecium and from *P. westbergii* in having less densely occurring apothecia and smaller spores.

##### Type.

Finland • Enontekiön Lappi, Enontekiö, Porojärvet, Toskalharji, Toska­lpahta, fell, dolomite rock outcrop, beneath SW-facing wall, on dolomite pebbles, 800 m a.s.l., 69°11'N, 21°30'E, 4 August 2011, J. Pykälä 43524 (H9250947 – holotype, GenBank accession number: PV766713).

##### Description.

Thallus white, grey or pale brown, rimose to areolate, areoles 0.15–0.4 mm, ca. 0.01–0.15 mm thick, K-, C-, UV-, algal cells 6–10 μm. Apothecia orange, 0.3–1.0 mm, convex to mainly strongly convex, superficial, high; ca. 20–40 apothecia / cm^2^, sometimes forming clusters of smaller apothecia on surface of old apothecia. Epihymenium orange-brown, 12–14 μm thick, K+ violet. Hymenium 60–80 μm thick. Hypothecium purple, orange-red to red-violet, ca. 100–280 μm thick. Paraphyses ca. 2–2.5(–3) μm thick, apex not thickened to slightly thickened, often branching. Ascospores 0-septate, (6.3–)7.9–9.2–10.5(–12.7) × (3.7–)4.2–4.6–5.1(–5.6) μm (n = 52).

##### Habitat and distribution.

The three specimens are known from the calcareous Toskalharji fell (NW Finland), all from dolomite pebbles. Companion species include *Farnoldia jurana* and *Lecidella stigmatea*.

##### Etymology.

The name refers to the violet hypothecium of the species.

##### Notes.

The species is related to *P. pseudoterricola* and *P. terricola*, which have mainly brown apothecia and broader spores. *Protoblastenia violacea* differs from *P. siebenhaariana* in having more densely occurring apothecia (only ca. 1–10 apothecia / cm^2^ in *P. siebenhaariana*; Kainz and Rambold (2004)), slightly smaller apothecia and a thinner hymenium. *Protoblastenia westbergii* has more densely occurring apothecia and larger spores.

##### Other specimens examined.

Finland • Enontekiön Lappi, Enontekiö, Porojärvet, Toskalharji, Toskalpahta, fell, SW-slope, on dolomite pebbles, 765 m a.s.l., 69°11'N, 21°29'E, 1.8.2011, J. Pykälä 43015 (H); • Enontekiö, Porojärvet, Toskalharji, 1.2 km NE of Toskaljärvi, fell, SW-slope, gentle E-slope, *Dryas* heath, on dolomite pebbles, 875 m a.s.l., 69°11'N, 21°30'E, 5.8.2011, J. Pykälä 43605 (H).

#### ﻿ Protoblastenia westbergii

Taxon classificationFungiLecanoralesPsoraceae

Pykälä & Myllys sp. nov.

A170C9C6-8A76-53FB-98BA-14A165D0344F

859663

Fig. 5D

##### Diagnosis.

Differs from *P. dolomitica* in having slightly broader spores, darker orange apothecia and a thicker hypothecium.

##### Type.

Finland • Enontekiön Lappi, Enontekiö, Kilpisjärvi, Saana Nature Reserve, E-part, fell, steep SW-slope, on dolomite boulder, 910 m a.s.l., 69°02'N, 20°51'E, 13 August 2011, J. Pykälä 44316 (H9229821 – holotype, GenBank accession number: PV766718).

##### Description.

Thallus white to whitish-grey, rimose to areolate, areoles 0.15–0.8 mm, ca. 0.05–0.3 mm thick, K-, C-, UV- to UV+ bluish-white, algal cells 4–8 μm, thallus often strongly invaded by cyanobacteria. Apothecia orange to dark brownish-orange, 0.2–1.1 mm, slightly convex to convex, 3/4-immersed to superficial, in one specimen with pale margin ca. 30–50 μm thick; ca. 40–200 apothecia / cm^2^. Epihymenium dirty yellow to orange-brown, 10–15 μm thick, K+ violet. Hymenium 40–70 μm thick, often with oil droplets. Hypothecium red-brown to red-violet, ca. 60–150 μm thick. Paraphyses ca. 2–2.5 μm thick, apex not thickened to slightly thickened to 3 μm thick, some branched and anastomosing. Ascospores 0-septate, (8.4–)10.5–12.0–13.6(–15.0) × (5.0–)5.4–6.1–6.9(–7.8) μm (n = 39).

##### Habitat and distribution.

Four specimens are known from the calcareous Saana and Toskalharji fells in NW Finland. On Saana, both specimens occurred close to each other on a steep SW-slope on a dolomite boulder. On Toskalharji, the species was found on a SE-facing slope on dolomite pebbles. The species may prefer dry, sun-exposed habitats. Companion species include *Farnoldia jurana*, *Parmeliella thriptophylla* (Ach.) Müll. Arg., *Thelidium auruntii* and *Verrucaria* sp.

##### Etymology.

The name is in honour of Dr Martin Westberg (Uppsala) for his important work on the taxonomy and biogeography of northern European lichens.

##### Notes.

The species is closely related to *P. dolomitica* (4% difference in ITS). *Protoblastenia dolomitica* has, on average, smaller and paler, sometimes olive-yellow apothecia, narrower spores and a thinner hypothecium. However, *P. westbergii* often also has a thin hypothecium. *Protoblastenia arupii* has slightly broader spores, a thin hypothecium and possibly thinner thallus.

##### Other specimens examined.

Finland • Enontekiön Lappi, Enontekiö, Porojärvet, Toskalharji, Toskaljärvi N, fell, dolomite scree, gentle SE-slope, on dolomite pebbles, 715 m a.s.l., 69°11'N, 21°26'E, 2.8.2011, J. Pykälä 43196 (H); • Enontekiö, Porojärvet, Toskalharji, Toskaljärvi N, fell, dolomite rock outcrop, SE-slope, on dolomite pebbles, 720 m a.s.l., 69°12'N, 21°26'E, 2.8.2011, J. Pykälä 43279 (H); • Enontekiö, Kilpisjärvi, Saana Nature Reserve, E-part, fell, steep SW-slope, on dolomite boulder, 910 m a.s.l., 69°02'N, 20°51'E, 13.8.2011, J. Pykälä 44314 (H).

### ﻿A preliminary key of *Protoblastenia* in Finland

**Table:** 

1	Apothecia K- or K+ weakly red	**2**
–	Apothecia K+ violet	**3**
2 (1)	Apothecia 0.2–0.6 mm, 40–100 apothecia / cm^2^, hemiboreal	***P. lilacina***
–	Apothecia 0.3–0.9 mm, 10–50(–80) apothecia / cm^2^, northern boreal	***P. oulankaensis***
3 (2)	Hypothecia violet to brown	**4**
–	Hypothecia white to yellow	**9**
4 (3)	ca. 20–40 apothecia / cm^2^	**5**
–	ca. 40–160 apothecia / cm^2^	**6**
5 (4)	Apothecia mainly brown, hypothecia 60–100 μm thick	***P. pseudoterricola***
–	Apothecia orange, hypothecia 100–280 μm thick	***P. violacea***
6 (4)	Thallus slightly rimose	***P. arupii***
–	Thallus rimose to areolate	**7**
7 (6)	Apothecia yellow, dirty orange yellow or olive yellow	***P. dolomitica***
–	Apothecia orange to brown orange	**8**
8 (7)	Apothecia convex to strongly convex, mean spore size 10.0 × 5.7 μm	***P. terricola***
–	Apothecia slightly convex to convex, mean spore size 12.6 × 6.3 μm	***P. westbergii***
9 (3)	Apothecia plane to slightly convex	**10**
–	Apothecia slightly convex to strongly convex	**11**
10 (9)	Hemi-boreal	***P. compressa***
–	Northern boreal	***P. pseudocompressa***
11 (9)	On lowlands	**12**
–	On calcareous fells	**17**
12 (11)	Apothecia convex to strongly convex, hypothecia 100–200 μm thick	**13**
–	Apothecia slightly convex to convex, hypothecia 40–160 μm thick	**15**
13 (12)	Thallus endolithic to sparse small areoles	***P. remota***
–	Thallus rimose to areolate	**14**
14 (13)	Apothecia orange-yellow to orange, 40–100 apothecia / cm^2^	***P. calvella***
–	Apothecia dirty brown-orange, 30–50 apothecia / cm^2^	***P. ekmanii***
15 (12)	Thallus mainly areolate, 0.1–0.2 mm thick	***P. rupestris***
–	Thallus mainly rimose, usually 0.02–0.1 mm thick	**16**
16 (15)	Apothecia plane to strongly convex, hymenia 50–60 μm thick	***P. pseudocompressa***
–	Apothecia slightly convex to strongly convex, hymenia 50–90 μm thick	***P. borealis***
17 (11)	Apothecia leave shallow to fairly deep pits, thallus endolithic	***P. minuta***
–	Apothecia not leave pits, rarely leave shallow pits, thallus usually epilithic	**18**
18 (17)	Hypothecia 60–300 μm thick	**19**
–	Hypothecia 40–100 μm thick	**20**
19 (18)	Apothecia convex to strongly convex, 20–40 apothecia / cm^2^	***P. rikkinenii***
–	Apothecia plane to strongly convex, 20–110 apothecia / cm^2^	***P. fennoarctica***
20 (18)	Apothecia 0.3–0.5 mm, mean spore size 8.4 × 4.8 μm	***P. saanaensis***
–	Apothecia 0.3–0.8 mm, mean spore size 9.2 × 4.8 μm	***P. timdalii***

## Supplementary Material

XML Treatment for Protoblastenia arupii

XML Treatment for Protoblastenia borealis

XML Treatment for Protoblastenia calvella

XML Treatment for Protoblastenia compressa

XML Treatment for Protoblastenia dolomitica

XML Treatment for Protoblastenia ekmanii

XML Treatment for Protoblastenia fennoarctica

XML Treatment for Protoblastenia lilacina

XML Treatment for Protoblastenia minuta

XML Treatment for Protoblastenia oulankaensis

XML Treatment for Protoblastenia pseudocompressa

XML Treatment for Protoblastenia pseudoterricola

XML Treatment for Protoblastenia remota

XML Treatment for Protoblastenia rikkinenii

XML Treatment for Protoblastenia rupestris

XML Treatment for Protoblastenia saanaensis

XML Treatment for Protoblastenia terricola

XML Treatment for Protoblastenia timdalii

XML Treatment for Protoblastenia violacea

XML Treatment for Protoblastenia westbergii

## ﻿Additional information

### Use of AI

No use of AI was reported.

### Author ORCIDs

Juha Pykälä https://orcid.org/0000-0002-7566-9310

Leena Myllys https://orcid.org/0000-0002-9566-9473

### Data availability

All of the data that support the findings of this study are available in the main text.

## References

[B1] Cannon P Aptroot A Coppins B Orange A Sanderson N Simkin J (2022) Lecanorales: Psoraceae, including the genera *Brianaria*, *Protoblastenia*, *Protomicarea* and *Psora*. Revisions of British and Irish Lichens 28: 1–11.

[B2] Castello M Nimis PL (1995) A critical revision of Antarctic lichens described by C. W. Dodge. Bibliotheca Lichenologica 57: 71–92.

[B3] Divakar PK Leavitt SD Molina MC Del-Prado R Lumbsch HT Crespo A (2016) A DNA barcoding approach for identification of hidden diversity in Parmeliaceae (Ascomycota): *Parmelia* sensu stricto as a case study. Botanical Journal of the Linnean Society 180: 21–29. 10.1111/boj.12358

[B4] Edgar RC (2004) MUSCLE: Multiple sequence alignment with high accuracy and high throughput. Nucleic Acids Research 32: 1792–1797. 10.1093/nar/gkh340 15034147 PMC390337

[B5] Ekman S Blaalid R (2011) The devil in the details: Interactions between the branch-length prior and likelihood model affect node support and branch lengths in the phylogeny of the Psoraceae. Systematic Botany 60: 541–561. 10.1093/sysbio/syr022 21436107

[B6] Evankow AM Yin A Zulfiqar R Ahmad UF Nordenhaug P Khalid AN Wang L Timdal E (2025) *Psora mediterranea* (Lecanorales, Psoraceae), a new lichen species from Europe, including a new concept for *P. himalayana* and a revised key to the European species. Mycological Progress 24: 26. 10.1007/s11557-025-02045-8

[B7] Gardes M Bruns TD (1993) ITS primers with enhanced specificity for basidiomycetes – application to the identification of mycorrhizae and rusts. Molecular Ecology 2: 113–118. 10.1111/j.1365-294X.1993.tb00005.x 8180733

[B8] Hafellner J (2006) *Protoblastenia szaferi* (lichenized Ascomycotina) – new to the Alps. Herzogia 19: 23–33.

[B9] Kainz C (2004) *Protoblastenia*. In: Nash III TH Ryan BD Diederich P Gries C Bungartz F (Eds) Lichen Flora of the Greater Sonoran Desert Region.Volume II. Lichens Unlimited, Arizona State University, Tempe, 424–425.

[B10] Kainz C Rambold G (2004) A phylogenetic study of the lichen genus *Protoblastenia* (Lecanorales, Psoraceae) in Central Europe. Bibliotheca Lichenologica 88: 267–299.

[B11] Lücking R Aime MC Robbertse B Miller AN Ariyawansa HA Aoki T Cardinali G Crous PW Druzhinina IS Geiser DM Hawksworth DL Hyde KD Irinyi L Jeewon R Johnston PR Kirk PM Malosso E May TW Meyer W Öpik M Robert V Stadler M Thines M Vu D Yurkov ANM Zhang N Schoch CL (2020a) Unambiguous identification of fungi: Where do we stand and how accurate and precise is fungal DNA barcoding? IMA Fungus 11: 1–32. 10.1186/s43008-020-00033-z PMC7353689 32714773

[B12] Lücking R Nadel MRA Araujo E Gerlach A (2020b) Two decades of DNA barcoding in the genus *Usnea* (Parmeliaceae): How useful and reliable is the ITS? Plant and Fungal Systematics 65: 303–357. 10.35535/pfsyst-2020-0025

[B13] Miadlikowska J Kauff H Högnabba F Oliver JC Molnár K Fraker E Gaya E Hafellner J Hofstetter V Gueidan C Otálora MAG Hodkinson B Kukwa M Lücking R Björk C Sipman HJM Burgaz AR Thell A Passo A Myllys L Goward T Fernández-Brime S Hestmark G Lendemer J Lumbsch HT Schmull M Schoch CL Sérusiaux E Maddison DR Arnold AE Lutzoni F Stenroos S (2014) A multigene phylogenetic synthesis for the class Lecanoromycetes (Ascomycota): 1307 fungi representing 1139 infrageneric taxa, 317 genera and 66 families. Molecular Phylogenetics and Evolution 79: 132–168. 10.1016/j.ympev.2014.04.003 24747130 PMC4185256

[B14] Munger I Baugh M Henrie JR Hollinger J Crepeau R Leavitt SD (2022) Integrative biodiversity inventories: Characterizing lichen-forming fungal diversity in Glen Canyon National Recreation Area using DNA barcoding and vouchered specimens. Western North American Naturalist 82(2): 213–233. 10.3398/064.082.0201

[B15] Myllys L Velmala S Holien H Halonen P Wang LS Goward T (2011) Phylogeny of the genus *Bryoria*. Lichenologist (London, England) 45: 617–638. 10.1017/S0024282911000132

[B16] Nowak J (1974) *Protoblastenia szaferi* sp. nov., a new lichen species in the calcareous part of the Polish Tatra Mts. Fragmenta Floristica et Geobotanica 20: 529–533.

[B17] Orange A (2012) Semi-cryptic marine species of *Hydropunctaria* (Verrucariaceae, lichenized Ascomycota) from north-west Europe. Lichenologist (London, England) 44: 299–320. 10.1017/S0024282911000867

[B18] Poelt J (1957) Mitteleuropäische Flechten. V. Mitteilungen der Botanischen Staatssammlung München 17–18: 386–399.

[B19] Printzen C Holien H Kantelinen A Myllys L Ratschow F Stepanchikova I Weber L Timdal E (2023) DNA barcoding indicates the presence of unrecognized species and phylogenetic diversity within the *Biatora vernalis*-and *B. meiocarpa*-groups. Plant and Fungal Systematics 68: 262–279. 10.35535/pfsyst-2023-0011

[B20] Pykälä J (2010) Notes on the lichen flora of Saana and Malla fells in northern Finland. Memoranda Societatis pro Fauna et Flora Fennica 86: 34–42.

[B21] Pykälä J (2023) Additions to the lichen flora of Finland. X. Graphis Scripta 35(3): 14–29.

[B22] Pykälä J Lommi S (2021) Lichen flora of Finland – short history of Finnish lichenology and updated species statistics. Memoranda Societatis pro Fauna et Flora Fennica 97: 73–88.

[B23] Pykälä J Launis A Myllys L (2017) Four new species of *Verrucaria* from calcareous rocks in Finland Lichenologist 49: 27–37. 10.1017/S0024282916000542

[B24] Pykälä J Kantelinen A Myllys L (2020) Taxonomy of *Verrucaria* species characterised by large spores, perithecia leaving pits in the rock and a pale thin thallus in Finland. MycoKeys 72: 43–92. 10.3897/mycokeys.72.56223 32963488 PMC7481264

[B25] Stamatakis A (2014) RAxML version 8: A tool for phylogenetic analysis and post-analysis of large phylogenies. Bioinformatics (Oxford, England) 30(9): 1312–1313. 10.1093/bioinformatics/btu033 24451623 PMC3998144

[B26] Stenroos S Velmala S Pykälä J Ahti T [Eds] (2016) Lichens of Finland. Norrlinia 30: 1–896.

[B27] Svensson M Ekman S Arup U Eide Ekman L Hammarström O Isaksson R Jonsson F Palice Z Vicente R Westberg M (2024) Further additions to the Swedish flora of lichenised fungi. Graphis Scripta 36(2): 15–49.

[B28] Timdal E (1987) Problems of generic delimitation among squamiform members of the Lecideaceae. In: Peveling E (Ed.) Progress and problems in lichenology in the eighties.Bibliotheca Lichenologica 25: 243–247.

[B29] White TJ Bruns T Lee S Taylor J (1990) Amplification and direct sequencing of fungal ribosomal DNA genes for phylogenetics. In: Innis MA Gelfand DH Sninsky JJ White TJ (Eds) PCR Protocols: a guide to methods and applications.Academic Press, San Diego, 315–322. 10.1016/B978-0-12-372180-8.50042-1

[B30] Wirth V Hauck M Schultz M (2013) Die Flechten Deutschlands. Band 2. Ulmer, Stuttgart, 1244 pp.

[B31] Zahlbruckner A (1936) Neue Flechten. XII. Annales Mycologici 34: 159–179.

[B32] Zhang Y Clancy J Jensen J McMullin RT Wang L Leavitt SD (2022) Providing scale to a known taxonomic unknown—At least a 70-fold increase in species diversity in a cosmopolitan nominal taxon of lichen-forming fungi. Journal of Fungi (Basel, Switzerland) 8: 490. 10.3390/jof8050490 PMC9146994 35628746

